# Production of real signs but not pseudosigns affected by age of acquisition in American Sign Language

**DOI:** 10.3758/s13421-024-01656-y

**Published:** 2025-01-22

**Authors:** Shai Lynne Nielson, Rachel I. Mayberry

**Affiliations:** 1 Department of Linguistics, University of California San Diego, 9500 Gilman Drive, La Jolla, CA 92093-0108, USA

**Keywords:** Language acquisition, Sign language, Phonological processing, Language production, Psycholinguistics

## Abstract

Research shows that insufficient language access in early childhood significantly affects language processing. While the majority of this work focuses on syntax, phonology also appears to be affected, though it is unclear exactly how. Here we investigated phonological production across age of acquisition of American Sign Language (ASL). Participants were deaf adult signers who first learned ASL at ages ranging from birth to 14 years and they performed both lexical decisions and repetitions of ASL signs and pseudosigns. Because phonological production has been understudied across age of acquisition, we were particularly interested in production accuracy for the sublexical phonological parameters of handshape, movement, and location. Lexical decision responses were slower and more accurate for impossible pseudosigns compared with possible pseudosigns, indicating participants were sensitive to ASL phonological structure regardless of age of acquisition. Despite this, age of acquisition affected repetition accuracy. Handshape errors were highest for those with earlier ages of acquisition, but movement errors were highest for those with later ages of acquisition, though this effect of age of acquisition was only seen for real ASL signs and not pseudosigns. The parameter error pattern for pseudosigns was not affected by age of acquisition. These results indicate that later age of acquisition does not inhibit the ability to produce ASL phonology but ultimately alters the processing of the phonological parameters when meaning and phonology are integrated.

## Introduction

While hearing children have immediate and automatic access to spoken language(s) used around them from birth, more than 90% of deaf children are born into hearing families and are unable to fully access any spoken language(s) in the environment, and they also have no access to any sign languages (Mitchell & Karchmer, 2004). These deaf children often receive spoken language instruction, speech therapy, and/or hearing devices to the exclusion of sign language exposure to promote speech. This approach is not uniformly successful, however, and consequently many of these deaf children do not receive full access to any language during the critical period for language in early childhood (Lenneberg, 1967; Mayberry & Kluender, 2018). These deaf children may eventually experience exposure to a sign language after early childhood, but research has shown differential outcomes for this unique circumstance of late language acquisition relative to early acquisition (Lillo-Martin & Henner, 2021; Mayberry & Kluender, 2018). Strong effects have been found for deaf signers with late age of acquisition (AoA) at the sentence-level (e.g., Mayberry et al., 2024), but less research has been devoted to investigating how late AoA signers^1^ process language at the phonological level. This kind of investigation is necessary as differences found in phonological processing could provide important insights into differences observed at higher levels of language processing. The goal of the present study was to investigate phonological processing as a function of AoA in American Sign Language (ASL) through lexical recognition and production of ASL signs and pseudosigns. Before describing the study, we turn to what is known about sign phonological processing in general and for late AoA signers specifically.

## Sign language phonological processing

Like all natural languages, sign languages have a phonological level composed of meaningless forms that come together to form units of meaning. While meaningless forms in spoken languages are organized into articulatory categories of consonants and vowels, meaningless forms in sign languages are organized into articulatory categories called parameters^2^—handshape, location, movement, and orientation. Every sign has a form specified for each of these parameters. For example, in ASL the citation form of the sign for PLEASE.^3^ has a B-handshape, a circular movement, a location on the chest, and an orientation of the palm facing the signer. Of the four parameters, handshape, location, and movement have received the most attention in phonological models and theories, providing the original evidence for dual patterning of meaningless, phonological forms that combine to create meaning in sign languages (Stokoe, 1960; Stokoe et al., 1965). This work began with the analysis of minimal pairs that showed that a difference in only one parameter form could change the meaning of a sign. For example, changing only the movement of the previously described sign PLEASE can change the meaning to create the sign for HAPPY. These kinds of analyses ultimately provide evidence for sign language phonology and for the manual forms being categorized into the sublexical parameters of handshape, location, movement, and orientation.

Psycholinguistic evidence for a sign language’s phonological system can be seen in everyday occurrences like signers’ slips of hand (Klima & Bellugi, 1979) and “tip of finger” experiences (Thompson et al., 2005), where, unconsciously, errors and lexical retrieval are guided by the differences in how each parameter is processed. In fact, movement, location, and handshape contribute differently to phonological structure, lexical access, and language processing more generally (see, e.g., Mayberry & Wille, 2022; Sandler & Lillo-Martin, 2006). Movement has been argued to be the syllable nucleus, like vowels in spoken languages, due to its dynamic and salient nature and necessity in sign syllables (see Brentari, 2019, for an overview). Location has been considered an organizing unit for the mental lexicon with inhibition effects seen when signs share a location (Baus et al., 2008; Carreiras et al., 2008; Corina & Hildebrandt, 2002). Electrophysiological evidence suggests that location’s unique contribution is at the lexical level as its effects are modulated by task and the need to engage lexical competition in identifying signs (Meade et al., 2021). Research investigating the contribution of handshape to sign processing through priming paradigms has produced mixed results (Carreiras et al., 2008; Corina & Hildebrandt, 2002; Dye & Shih, 2006). However, handshape may have a special contribution in the creation and learning of lexical items due to its potential iconicity in form-meaning mappings (Occhino et al., 2017, 2020).

Many studies have looked at how combinations of parameters influence sign processing as well. These studies have investigated effects of each combination of handshape, movement, and/or location (e.g., Thompson et al., 2013; Wienholz et al., 2021), but the unit of movement and location has consistently been shown to have a prominent place in the phonological system of early AoA signers who acquired a sign language early in childhood (also referred to as native signers). Early AoA signers judge sign similarity primarily based on shared movement and location (Hall et al., 2012; Hildebrandt & Corina, 2002), and their lexical access is facilitated by shared movement and location (Baus et al., 2008; Corina & Knapp, 2006; Dye & Shih, 2006). This movement and location unit has been argued to be a syllable unit in sign languages and plays a key role in sign language phonological models (e.g., Brentari, 1998; Perlmutter, 1993; Wilbur, 1987).

There are also differences in how the parameters are acquired. Early AoA signers have been shown to follow specific patterns in phonological development, such as acquiring location first, then movement, and then handshape, with young children making the most errors in handshape, followed by movement, and then location (Conlin et al., 2000; Marentette & Mayberry, 2000; Siedlecki & Bonvillian, 1993). These patterns correspond with motor development in children as location accuracy relies primarily on gross motor skills that develop early in childhood while handshapes require fine motor skills that develop later.

Evidence indicates that hearing second language (L2) signers who acquired a spoken language in childhood and then learned a sign language as an L2 in adulthood show similar phonological patterns to early AoA signers. Hearing L2 signers can reach levels of phonological fluency comparable with deaf early AoA signers with enough exposure and language use (Beal & Faniel, 2019). They perform similarly to deaf early AoA signers on tasks consisting of sign recognition (Morford & Carlson, 2011) and sign similarity judgments (Hall et al., 2012). Hearing L2 signers also appear to show the same pattern of parameter errors as early AoA signers with the most errors in handshape, then movement, and then location (Ortega & Morgan, 2010, 2015).

## Late age of acquisition and phonological processing

While both deaf early AoA and hearing L2 signers have full language access early in childhood regardless of the modality, there are the aforementioned late AoA signers who do not have full access to any language early in childhood. More than 90% of deaf children are born into hearing families, and many of them do not have access to a sign language during early childhood because it is absent from the environment, and they are also unable to fully access any spoken languages used around them (Mitchell & Karchmer, 2004). These deaf signers eventually acquire a sign language after early childhood, but the context in which these late AoA signers acquire that language is an important factor.

Compared with infants, late AoA signers are older and have greater motor control, cognitive maturity, and world knowledge. This developmental circumstance differs significantly from the situation of infants who acquire language in concert with motor control and world knowledge. This difference of context could be seen as providing an advantage to late AoA signers (e.g., Newport, 1988) since they show faster vocabulary acquisition and an earlier two-word stage after exposure than young children acquiring a language from birth (Ferjan Ramírez et al., 2013). However, it is more likely to be a disadvantage because infants benefit from acquiring language with constraints on motor and cognitive development (e.g., Kuhl, 2004). This is supported by dramatically different language outcomes for late AoA signers both behaviorally and neurolinguistically compared with early AoA signers (e.g., Cheng et al., 2023) and hearing L2 signers (see Mayberry & Kluender, 2018, for an overview). Much research has focused on sentence-level processing for late AoA signers, though studies focused on the phonological level indicate that late AoA signers also have differential outcomes for phonology.

Compared with early AoA signers, late AoA signers have been shown to make more phonological than semantic errors in shadowing tasks (Mayberry & Fischer, 1989) and make similarity judgments of signs that pattern more like those of nonsigners (Hall et al., 2012). Late AoA signers also show less categorical organization of phonemic representation than early AoA signers (Best et al., 2010; Morford et al., 2008). While early AoA signers show facilitation effects in sign recognition when signs share both movement and location (Dye & Shih, 2006; Thompson et al., 2013), late AoA signers show more facilitation for shared handshape (Wienholz & Lieberman, 2020). Late AoA signers also show an overreliance on handshape in sign monitoring (Morford & Carlson, 2011) and similarity judgment tasks (Hildebrandt & Corina, 2002). In the only study to look at phonological development, Morford (2000) found that two case studies who acquired ASL in adolescence demonstrated high levels of handshape production accuracy within their first year of ASL exposure, though the other parameters were not investigated. It is unclear why late AoA signers may give greater attention to handshape over the other sign parameters, but it is becoming clearer that how they process the parameters differs from that of early AoA signers who attend to movement and location.

## Current study

While there is growing evidence that late AoA may lead to a reliance on handshape in sign recognition and perception, phonological production of all the sign parameters has not been analyzed across AoA. To address this gap, we investigated the effects of AoA on parameter production in ASL.

Prior to a production task, participants completed a lexical decision task. Lexical decisions require participants to be sensitive to ASL phonotactics and lexicality (i.e., the rules governing ASL phonology). While it may be expected that longtime users of a language would have knowledge of their language’s phonological structure, this is something that needed to be tested for late AoA signers. As discussed in the Introduction, late AoA signers have dramatically different language outcomes compared with early AoA signers (e.g., Lillo-Martin & Henner, 2021; Mayberry & Kluender, 2018), and little is known about their phonological processing. It was possible that any effects of AoA observed in phonological productions could be caused by a lack of sensitivity to ASL phonological structure, which could be seen in AoA effects on lexical decisions. Our hypothesis for the lexical decision task was that we would not see effects of AoA on lexical decision response time or accuracy and that all signers regardless of AoA would show sensitivity to ASL phonology, but it was crucial to confirm this before interpreting any results related to the production of ASL phonology.

Following the lexical decision task, participants completed a lexical repetition task which allowed us to investigate response time, production time, and production accuracy of signers across AoA for real signs and pseudosigns. We expected effects of lexicality on response time and production time such that pseudosigns would have longer response times and production times compared with real signs as they were novel forms that would need more processing time. We did not expect response time and production time to be affected by AoA, but we did test this, as AoA effects on either could potentially contribute to any effects observed in production accuracy, the measure where we did expect AoA effects.

Overall, we predicted that late AoA signers would have more phonological errors than early AoA signers. For real signs, we predicted that parameter errors would differ such that late AoA signers would have less handshape errors and early AoA signers would have less movement and location errors due to each group relying more on those respective parameters in sign processing. For pseudosigns, though, there were two possibilities. If late AoA signers diverged from early AoA signers for both real signs and pseudosigns, that would be evidence that late AoA signers may not be capable of producing phonological forms in the same ways as early AoA signers. However, if late AoA signers produce pseudosigns similarly to early AoA signers, that would be evidence that late AoA signers are capable of producing ASL phonological forms like early AoA signers but process those forms differently when meaning is integrated.

In short, we hypothesized that late AoA would alter real sign parameter errors and that the results of the pseudosign repetitions would inform us as to whether the effect was due to the phonological capabilities of late AoA signers or due to altered phonological processing when meaning is involved. Together, the results would provide insight into how late AoA signers process language and phonology specifically.

## General methods

### Participants

Sixty-seven adults who were born severely to profoundly deaf (≥ 80 dB pure-tone average in the better ear confirmed by audiometric testing) volunteered for the study. All but three participants, whose data were not used, scored within the normal range on a nonverbal cognitive screening task. One participant was excluded for not following task instructions. The remaining 63 participants (31 women, 32 men) had used ASL as their preferred language for 29 years on average (range: 8–59 years) and had an average age at testing of 35 years (range: 19–59 years old). Age of acquisition of ASL ranged from birth to 14 years and was operationalized as the age when the participant first began to learn ASL in an immersion setting (e.g., the home for early learners or school for later learners). Of the 63 participants, 32 reported using hearing aids at some point during childhood and only four continued to do so sometimes. Of the 29 participants who reported no longer using hearing aids, the age at which they stopped ranged from 4 to 21 years, with the primary reason being that hearing aids did not help them.

The participants with later AoAs were originally enrolled in mainstream educational programs where sign languages were either prohibited or where they were the only deaf child in a classroom of hearing children.^4^ In these education settings, participants received explicit instruction in English reading and writing in addition to speech therapy. Their educational programs were later changed to ones using sign language for classroom instruction and education after inadequate success with a strictly auditory–oral approach to communication. Their spoken language exposure likely led to some degree of learning spoken phonology prior to learning ASL; therefore, if the results of this study follow our hypothesis that late AoA signers process phonology differently from early AoA signers, the results will be despite any potential amounts of insufficient English acquired prior to ASL exposure.

For this study, sample size was not determined by a priori power analysis but instead by availability of participants. Deaf infants who have access to a sign language from birth constitute less than 10% of the overall deaf population (Mitchell & Karchmer, 2004). Similarly, those who are exposed to a sign language after birth vary greatly in the age when the exposure occurs. For these reasons, recruiting deaf signers across every AoA can be difficult. The aim for this study was to recruit an even number of participants from each of three AoA groups: 0–3 years, 4–8 years, and 9–14 years. This resulted in 22 participants in the 0–3 group, 20 participants in the 4–8 group, and 21 participants in the 9–14 group. For the current study, analyses were done across AoA and not by group, with the number of participants per AoA shown in Table 1.

Participants were asked to self-assess their language skills on a scale of 0 (*not at all*) to 10 (*perfect*) for ASL comprehension, ability to understand the speech of friends, and the ability to understand the speech of strangers. We used linear regression models to test the effect of AoA on the continuous variable of each self-assessed skill rating. There was no effect of AoA on self-rating of ASL comprehension (mean = 8.8, range: 5–10; β = − 0.064, *SE* = 0.036, *p* = 0.079), ability to understand the speech of friends (mean = 3.0, range: 0–9; β = 0.143, *SE* = 0.076, *p* = 0.066), nor the ability to understand the speech of strangers (mean = 2.4, range: 0–9; β = 0.055, *SE* = 0.062, *p* = 0.38). These results indicate that participants regarded their ASL proficiency as being high and their speech comprehension proficiency as being low, regardless of AoA. Because of this, we can conclude that participants were not able to reliably depend on audition for language acquisition and any effects found in this study are not due to differences in auditory access to spoken languages.

### Stimuli

The stimuli were 288 sign pairs consisting mostly of nouns and adjectives. The stimuli included no fingerspelled items or classifiers. Handedness (one vs. two hands used in a sign) was not controlled in sign pairs. Videos of all stimuli are available online (https://osf.io/n23wh/) and lists of the stimuli are available in Appendix 1, Tables 9, 10, 11.

#### Real signs

The stimuli consisted of prime and target sign pairs. All primes were ASL signs and half of the targets were also real ASL signs. Of the real ASL target signs, 72 were completely unrelated to the prime sign while the other 72 pairs were either phonologically related, semantically related, or their English glosses rhymed. In the current study, our main question is centered on phonological production, so we included only the unrelated real sign trials in the following analyses since the related trials address questions about lexical processing that are beyond the scope of the current study. Ultimately, the related trials did not reveal significant results, so their analyses are discussed in the Supplemental Materials.

Mayberry et al. (2014) asked the same participants as this study to provide subjective frequency ratings to 432 ASL signs on a scale ranging from 1 (*rarely see the sign*) to 7 (*always see the sign*). Subjective frequency ratings and response times did not vary as a function of the participants’ AoA of ASL, chronological age, or length of ASL experience (Mayberry et al., 2014). A subset of those signs are the real signs that appear in the current study and the difference in subjective frequency ratings between primes and targets was low (mean difference = 0.09, range: 0–3.83).

#### Pseudosigns

The other half of the targets were pseudosigns with no meaning in ASL. The pseudosigns were created by altering one parameter (handshape, location, or movement) of an ASL sign (a common method in sign language studies; e.g., Banaszkiewicz et al., 2021; Caselli et al., 2021; Emmorey et al., 2011), with an equal number of alterations across all three parameters across the pool of pseudosigns. Of the pseudosigns, 72 were possible ASL signs where the altered parameter came from the inventory of attested ASL features for each parameter. The single altered parameter resulted in a pseudosign that was consistent with ASL phonotactics. In contrast, the other 72 were impossible ASL lexical items because the altered parameter violated ASL phonotactics, such as a handshape, movement, or location not found in the ASL inventory or a violation of the symmetry and dominance rules for two-handed signs (Battison, 1978). After creating a set of potential pseudosigns, four native signers independently judged the pseudosigns by answering two questions: (1) is the item an ASL sign, and (2) is the pseudosign a possible ASL sign. The final set of pseudosigns included only those items for which the signers gave unanimous judgments to both questions. In the signpseudosign trials, there was no lexical-structural relationship between the prime and target signs.

#### Stimulus creation

A deaf native ASL signer produced each real sign and pseudosign several times with neutral facial expression and no mouthing while being videotaped. The renditions were judged by three other native signers, and those that represented the clearest sign production were selected as stimuli and edited into a series of individual video clips. The recognition point of each stimulus sign was then identified and defined as the video frame within which a sign’s production was complete (i.e., when all the parameters were in place). Each video clip was edited so that it began when the signer’s hands could be detected to transition into the stimulus sign and ended when the hands could be detected to transition out of the sign. Each video clip was edited so that an equal number of video frames preceded and followed the recognition point of the stimulus sign. Mean duration of the stimuli was eight video frames at 30 fps, or approximately 267 ms, and this did not differ across the conditions.

#### Trial structure

Half of the trials had a 300-ms interstimulus interval (ISI) between the primes and targets, and the other half had a 1,000-ms ISI. The ISI between primes and targets has been shown to influence reaction times, with longer ISIs providing more processing time and reduced priming effects compared with shorter ISIs where priming effects are more readily observed (e.g., Hildebrandt & Corina, 2002). This was primarily of interest for investigating priming in the related real sign trials (Supplemental Materials). However, both ISIs were also used in the unrelated real sign trials and the pseudosign trials and are thus included as variables in the analyses of the current study.

**Fig. 1:**
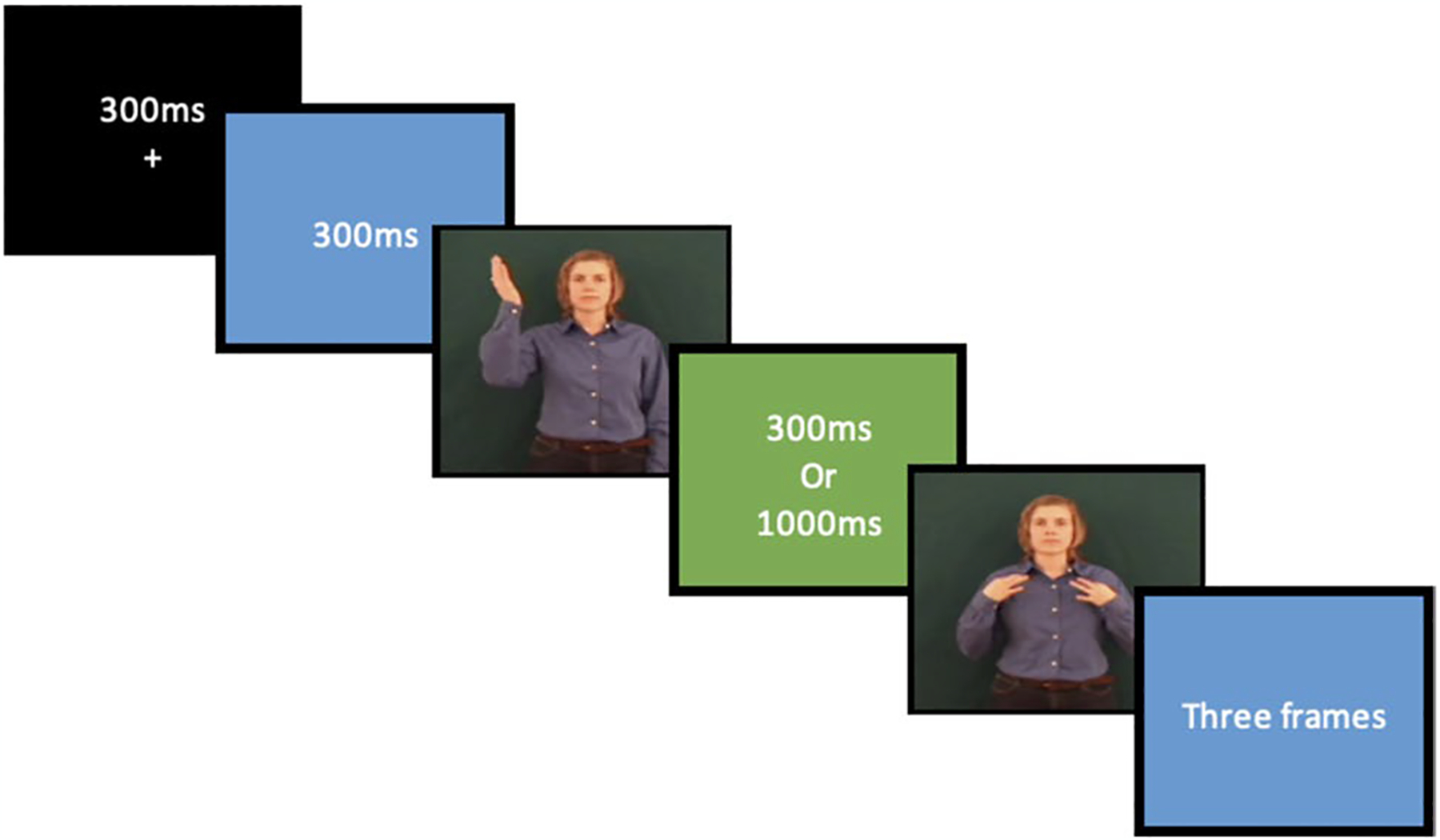
Example of the structure of each trial for both the lexical decision and repetition tasks

Consequently, each trial had the following structure: (1) a focus mark (+) appeared on the screen for 300 ms followed by (2) 300 ms of blue screen, then (3) the prime sign appeared dynamically (4) followed by a green screen of either 300 ms or 1,000 ms duration; (5) the target sign appeared next, followed by (6) three frames of blue screen (Fig. 1).

### Procedure

#### Experimental setup

Stimuli were presented on an Apple PowerBook G3 computer using PowerLaboratory software (Chute & Daniel, 1996), which recorded response time and accuracy for the lexical decision task and response time for the repetition task. For the lexical decision task, the participants held a game pad in their hands, indicating “yes” with the right thumb and “no” with the left. For the repetition task, participants rested their hands on a spring-loaded metal level placed over the space bar on the keyboard. A key press occurred when the participants lifted their hands to produce the target stimulus thereby recording response time to repeat a target sign. The amount of time required for participants to produce a sign (production time) was calculated as the time between removing their hands from the metal lever and then returning them after the production. We expected production time to be affected by lexicality such that pseudosigns would take longer to be produced than real signs, as they were novel and unfamiliar to all participants. We also did not expect AoA to affect production time but wanted to confirm that before analyzing production accuracy. A video camera recorded the participants’ sign productions for that later accuracy transcription.

#### Testing procedure

Research assistants who were deaf and experienced ASL signers tested the deaf participants. Each participant was tested individually in a non-distracting room. The McGill University Faculty of Medicine IRB approved the protocol. Informed consent was obtained through ASL, and participants were compensated for their time.

## Experiment 1: Lexical decision

All participants completed the lexical decision task first. Again, this task assessed participants’ knowledge of ASL’s phonological structure through their sensitivity to ASL phonotactics and lexicality prior to the repetition task assessing their productions of that structure. This task included all 288 trials of the sign pairs, and the pairs were randomized and grouped into four blocks of 72 trials. Block presentation order was counter-balanced across the participants with a Latin-squares design. All participants were told that they would see pairs of signs, some including signs that do not exist in ASL, and that they were to decide with a button press whether the target (second) sign was an ASL sign, yes or no. They began the lexical decision experiment only after demonstrating that they understood the task with a practice block of 20 pairs not included in the experiment. Participants were allowed to take breaks in between the experimental blocks, but none did so.

The lexical decision data were analyzed for response time and accuracy of decisions and the 72 unrelated real sign trials, 72 possible pseudosign trials, and 72 impossible pseudosign trials are included in the following analyses. Analyses of the related trials are in the Supplemental Materials. All models were fit using the lme4 (Version 1.1–26; Bates et al., 2015), lmerTest (Version 3.1–3; Kuznetsova et al., 2017), and car (Version 3.1–2; Fox & Weisberg, 2019) packages in R (R Core Team, 2021). All analyses were conducted with a threshold for significance of alpha = 0.01. When the variable of AoA was replaced by age at testing or years of experience in the following models, there was no effect of either variable.

### Results

#### Response time

We used linear mixed-effects modeling (lmer) to test the fixed effects of AoA, ISI, and trial condition (real sign, possible pseudosign, impossible pseudosign), along with interactions between the effects, on the continuous outcome variable of response time in correct trials, with random intercepts for participants and trial. Sum coding was used for the two-level contrasts of ISI (300 ms = + 1, 1,000 ms = − 1) and condition (possible pseudosigns = + 1, real signs = − 1; impossible pseudosigns = + 1, real signs = − 1). Three participants did not have response times recorded due to equipment failure for the lexical decision trials, so they were excluded from response time analyses for the lexical decision task.

**Fig. 2:**
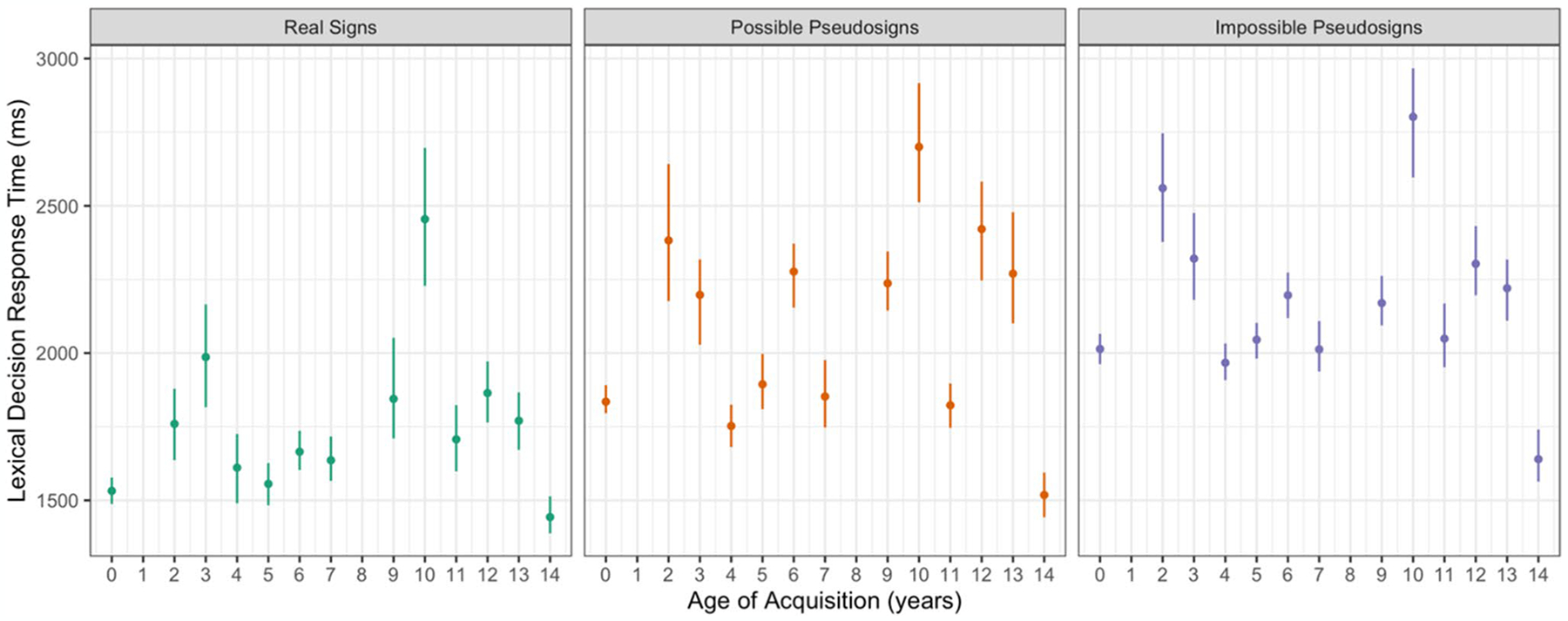
Lexical decision response time for correct trials by trial condition and AoA with 95% confidence intervals

Participants took longer to respond to possible pseudosign targets (β = 207.709, *SE* = 33.728, *p* < 0.001) and impossible pseudosign targets (β = 383.483, *SE* = 33.260, *p* < 0.001) compared with the real sign targets (Fig. 2).^5^ There were no main effects of AoA or ISI, nor any interactions between any of the variables (Table 2). This result shows that participants were sensitive to ASL phonotactics and lexicality regardless of AoA.

To directly compare the impossible pseudosigns with the possible pseudosigns, we again used linear mixed-effects modeling (lmer) to test the fixed effects of AoA, ISI, and trial condition (possible pseudosign, impossible pseudosign), along with interactions between the effects, on the continuous outcome variable of response time in correct pseudosign trials only, with random intercepts for participants and trial. Sum coding was used for the two-level contrasts of ISI (300 ms = + 1, 1,000 ms = − 1) and condition (impossible pseudosigns = + 1, possible pseudosigns = − 1).

Participants had longer response times for the impossible pseudosigns than the possible pseudosigns (β = 170.196, *SE* = 75.917, *p* = 0.002; Fig. 2). There were no main effects of AoA or ISI, nor any interactions between any of the variables. Not only were participants slower to respond to both kinds of pseudosign targets than the real sign targets, but they were slower still responding to the impossible pseudosign targets that violated ASL phonotactic structure than the possible pseudosigns. This shows that the participants, independent of AoA, were sensitive to ASL phonological structure.

#### Accuracy

We used generalized binomial linear mixed-effects modeling (glmer) to test the fixed effects of AoA, ISI, and trial condition (real sign, possible pseudosign, impossible pseudosign), along with interactions between the effects, on the binary variable of lexical decision accuracy (0 = correct decision, 1 = incorrect decision), with random intercepts for participants and trial. Sum coding was used for the two-level contrasts of ISI (300 ms = + 1, 1,000 ms = − 1) and condition (possible pseudosigns = + 1, real signs = − 1; impossible pseudosigns = + 1, real signs = − 1).

**Fig. 3:**
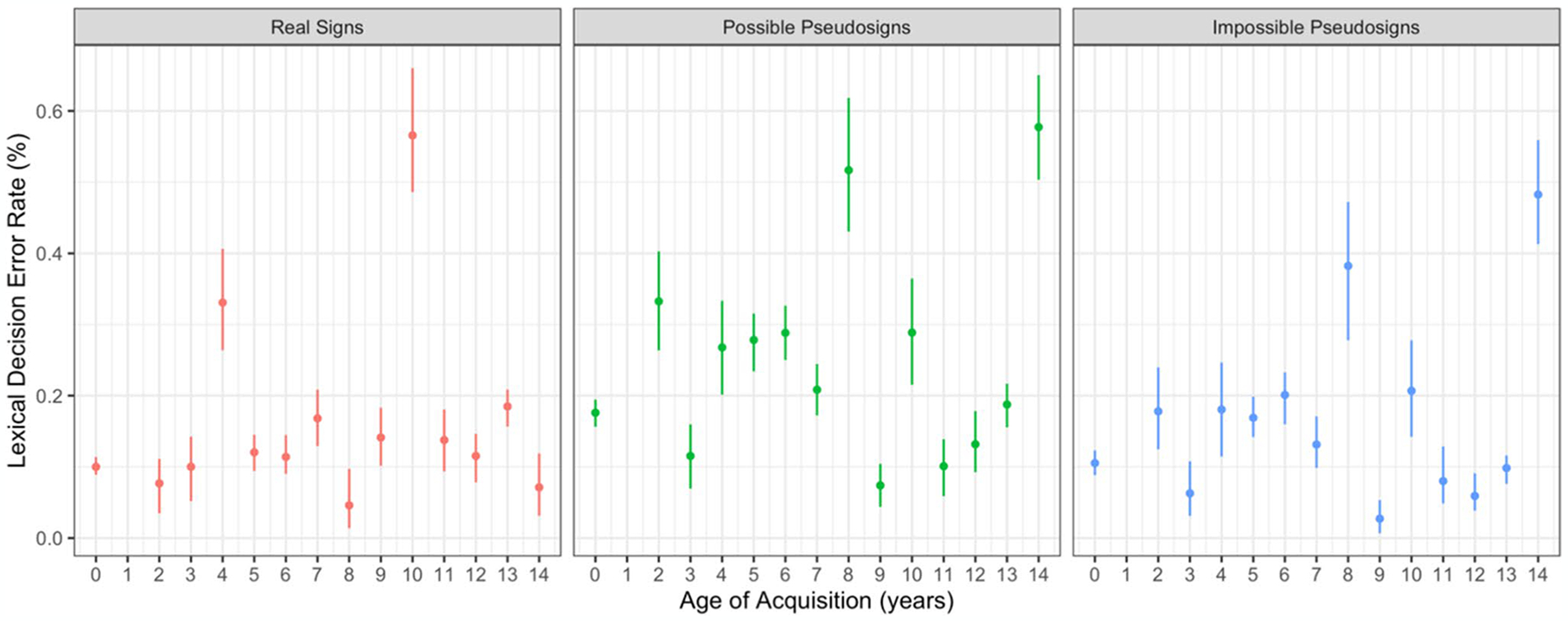
Lexical decision error rate by trial condition and AoA with 95% confidence intervals

Participants made more errors in response to possible pseudosigns compared with real signs (β = 0.783, *SE* = 0.134, *p* < 0.001; Fig. 3). There were no main effects of AoA or ISI, nor any interactions between any of the variables (Table 3).

To again directly compare the impossible pseudosigns with the possible pseudosigns, we used linear mixed-effects modeling (lmer) to test the fixed effects of AoA, ISI, and trial condition (possible pseudosign, impossible pseudosign), along with interactions between the effects, on the binary variable of lexical decision accuracy (0 = correct decision, 1 = incorrect decision), with random intercepts for participants and trial. Sum coding was used for the two-level contrasts of ISI (300 ms = + 1, 1,000 ms = − 1) and condition (impossible pseudosigns = + 1, possible pseudosigns = − 1).

Participants made less errors in response to impossible pseudosigns compared with possible pseudosigns (β = − 0.729, *SE* = 0.206, *p* < 0.001; Fig. 3). There were no main effects of AoA or ISI, nor any interactions between any of the variables. These two accuracy results show that possible pseudosigns were more often mistaken for real signs, but phonotactically impossible pseudosigns were not. Again, this provides support that participants were sensitive to ASL phonotactics and phonological structure regardless of AoA.

### Lexical decision summary

We analyzed lexical decision response time and accuracy for ASL signers of varying AoAs. Possible pseudosigns and impossible pseudosigns elicited longer response times compared with real signs across AoA. Additionally, impossible pseudosigns that violated ASL phonological structure were recognized as not being real signs more accurately and more slowly than possible pseudosigns. Altogether the lexical decision results indicate that participants were sensitive to ASL phonological structure regardless of AoA, allowing us to investigate how the signers produce that structure.

## Experiment 2: Lexical repetition

All participants completed the lexical repetition task after the lexical decision task. This task included 216 sign pairs from the real sign conditions and the possible pseudosign condition. The impossible pseudosign trials were excluded from the repetition task with the reasoning that while the ability to distinguish legal from illegal phonological forms in ASL would be informative, the ability to repeat illegal forms was beyond the scope of the current study. The sign pairs were randomized and grouped into four blocks of 54. Block presentation order was counterbalanced across the participants with a Latin-squares design. All participants were told that they would see pairs of signs, some including signs that do not exist in ASL, and that they were to repeat the second sign of each pair exactly as the signer in the video signed it. Participants were instructed to place their hands on the metal lever until they were ready to produce a sign. They began the repetition task after demonstrating that they understood it with a practice trial of 12 pairs not included in the experiment. Participants were allowed to take breaks in between the experimental blocks, but none did so.

The original videotapes of each participant’s repetition performance along with the stimulus videos were imported into the linguistic annotation software ELAN (Crasborn et al., 2006). Repetition performance was transcribed independently by two deaf native signers highly experienced with ASL transcription. They were instructed to determine accuracy based on whether the repetitions were exact copies of the target signs, as that was the instruction for participants. They noted correct and incorrect repetitions of the target signs by each phonological parameter. The transcribers agreed on 94% of the 28,944 total repetition trials they analyzed. In the case of disagreement, the final decision of the second transcriber was used for analysis.

Handshape errors were predominantly a change in handshape (e.g., W instead of V in TO-SMOKE, R instead of N in NIECE) and were not due to allophonic variation (e.g., open-B or closed-B for BIRTH). Movement errors included changes in type (e.g., circular instead of wrist twist for PURPLE, drag up-and-down instead of tap for CANADA) and changes in direction (e.g., backwards instead of forwards in TYPE/KIND). Location errors included lowered locations (e.g., start at cheek instead of forehead for MAN) and changes in major location (e.g., shoulder instead of under chin for DIRTY). There were fewer orientation errors and the changes varied (e.g., palms downward instead of sideways in COURT; palm upward instead of to the side in THROW).

The lexical repetition data were analyzed for response time, production time, and accuracy of repetition, with the 72 unrelated real sign trials and the 72 possible pseudosign trials discussed in the following analyses. Analyses of the related trials are in the Supplemental Materials. All models were fit using the lme4 (Version 1.1–26; Bates et al., 2015), lmerTest (Version 3.1–3; Kuznetsova et al., 2017), and car (Version 3.1–2; Fox & Weisberg, 2019) packages in R (R Core Team, 2021). All analyses were conducted with a threshold for significance of alpha = 0.01. When the variable of AoA was replaced by age at testing or years of experience in the following models, there was no effect of either variable.

### Results

#### Response time

We used linear mixed-effects modeling (lmer) to test the fixed effects of AoA, ISI, and trial condition (real sign, possible pseudosign), along with interactions between the effects, on the continuous outcome variable of response time in correct trials, with random intercepts for participants and trial. Sum coding was used for the two-level contrasts of ISI (1,000 ms = + 1, 300 ms = − 1) and condition (possible pseudosigns = + 1, real signs = − 1).

**Fig. 4:**
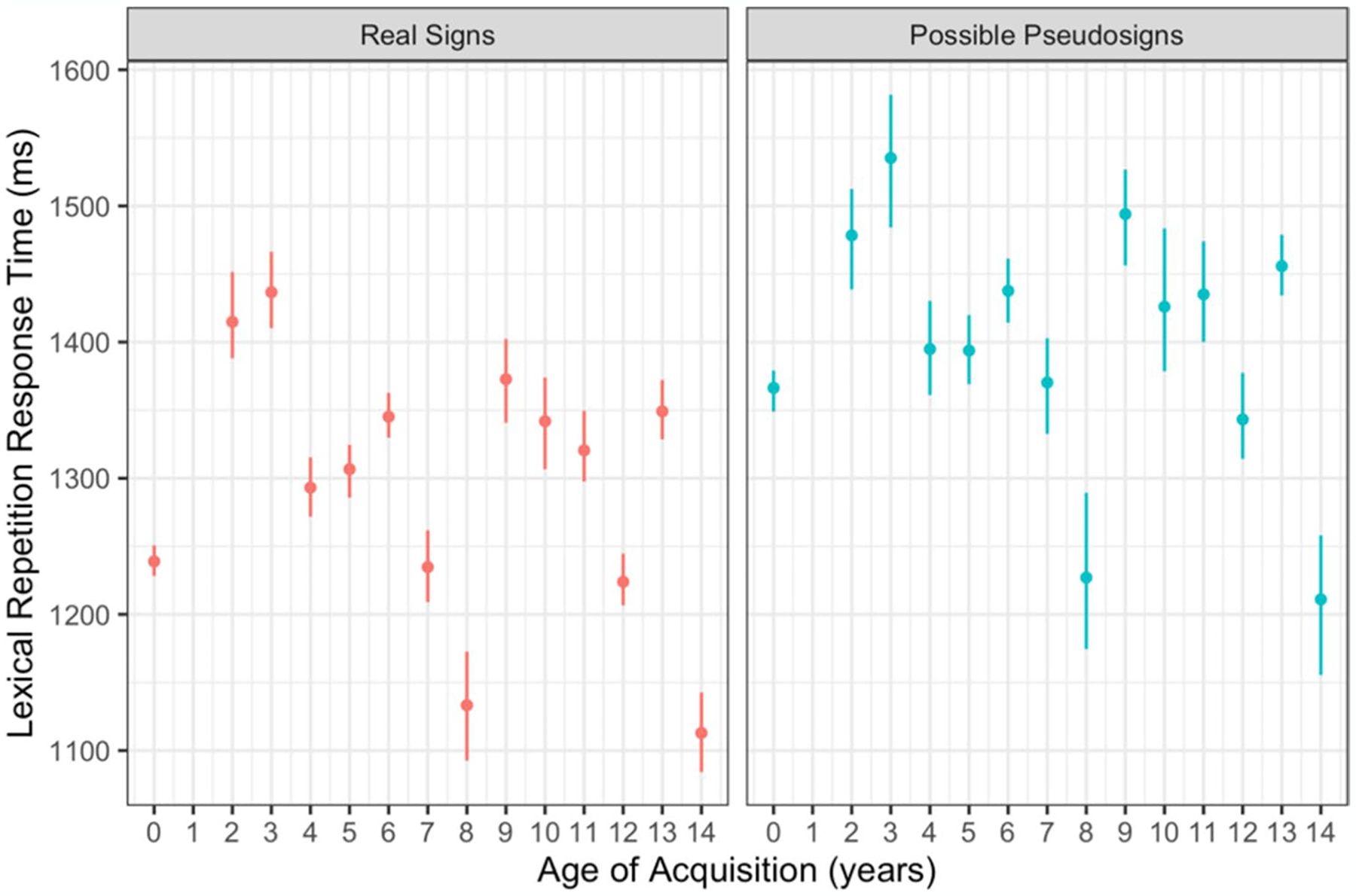
Lexical repetition response time by trial condition and AoA with 95% confidence intervals

Participants had longer response times for possible pseudosign targets compared with real sign targets (β = 124.179, *SE* = 19.118, *p* < 0.001; Fig. 4). There were no main effects of AoA or ISI, nor any interactions between any of the variables (Table 4). This result was expected as participants likely had to search longer through their mental lexicon to determine whether a pseudosign was a real sign or not, regardless of AoA.

#### Production time

We used linear mixed-effects modeling (lmer) to test the fixed effects of AoA, ISI, and trial condition (real sign, possible pseudosign), along with interactions among the effects, on the continuous outcome variable of production time in correct trials, with random intercepts for participants and trial. Sum coding was used for the two-level contrasts of ISI (1,000 ms = + 1, 300 ms = − 1) and condition (possible pseudosigns = + 1, real signs = − 1). For two participants, an error in gathering production time for a number of their trials resulted in excluding them from these analyses.

**Fig. 5:**
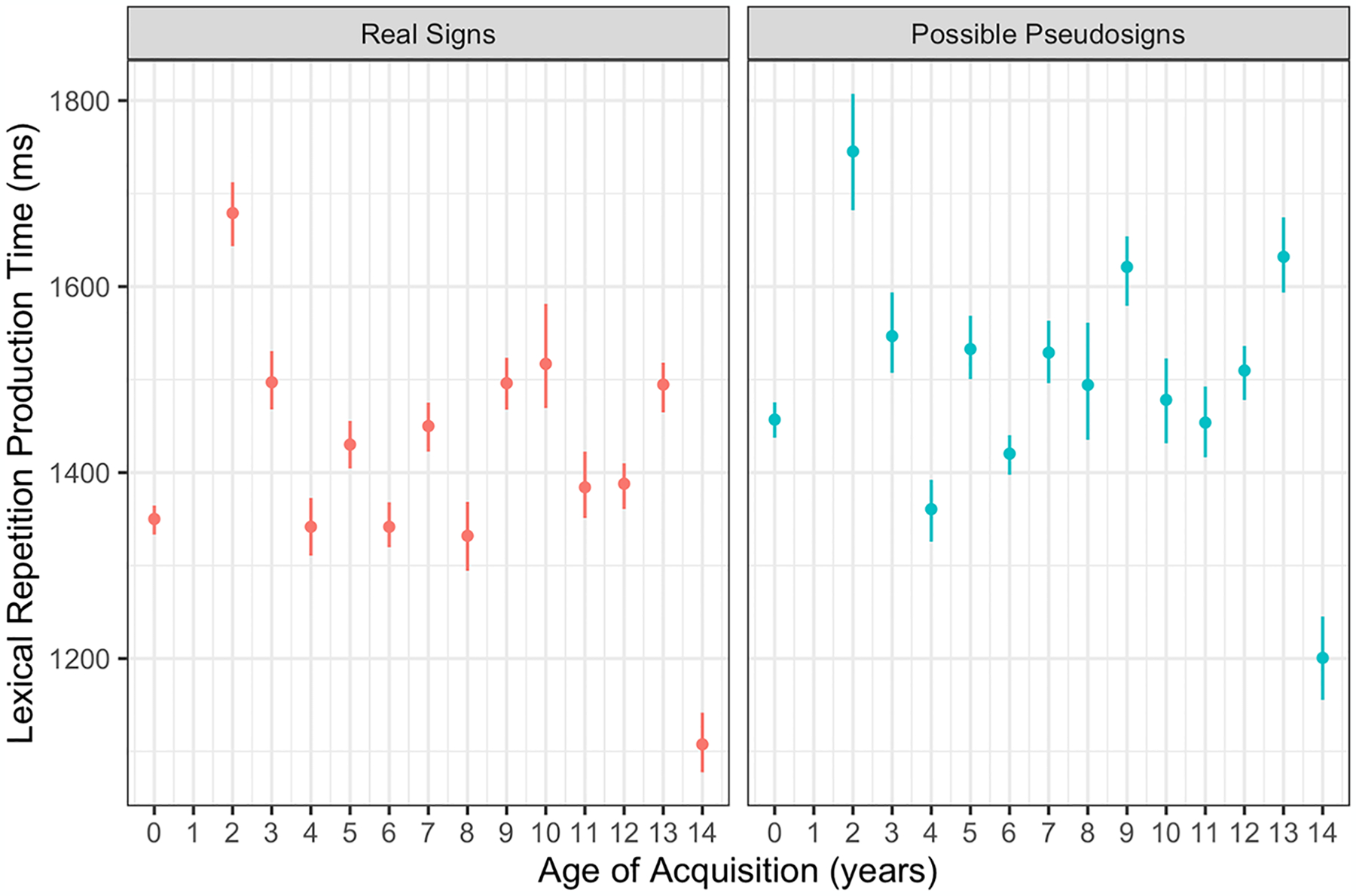
Lexical repetition production time by trial condition and AoA with 95% confidence intervals

Participants had longer production times for possible pseudosign targets compared with real sign targets (β = 78.617, *SE* = 13.359, *p* < 0.001; Fig. 5). There were no main effects of AoA or ISI, nor any interactions between any of the variables (Table 5). This lexicality effect was again expected as participants had never produced the pseudosigns before and likely needed more time to process and produce the novel forms. This result also shows that participants had similar rates of signing regardless of AoA, providing evidence that AoA did not affect production speed even if it did affect production accuracy, discussed below.

#### Trial accuracy

We used generalized binomial linear mixed-effects modeling (glmer) to test the fixed effects of AoA, ISI, and trial condition (real sign, possible pseudosign), along with interactions between the effects, on the binary variable of lexical repetition accuracy (0 = no error in repetition, 1 = at least one phonological error in repetition), with random intercepts for participants and trial. Sum coding was used for the two-level contrasts of ISI (1,000 ms = + 1, 300 ms = − 1) and condition (possible pseudosigns = + 1, unrelated real signs = − 1).

**Fig. 6:**
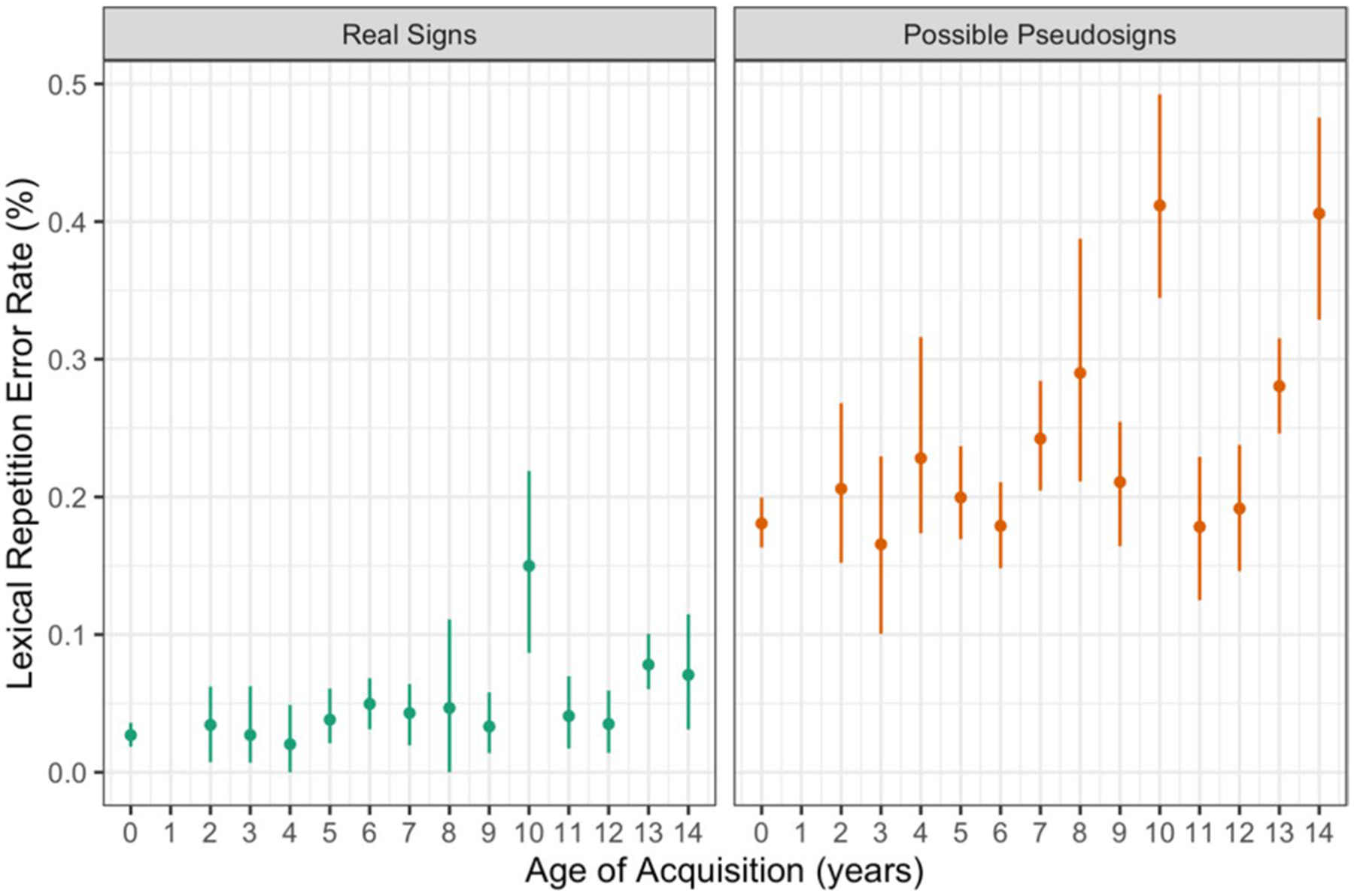
Lexical repetition error rate by trial condition and AoA with 95% confidence intervals

Participants made more repetition errors on the pseudosign targets than the real sign targets (β = 2.041, *SE* = 0.181, *p* < 0.001) and those with later AoAs made more repetition errors overall (β = 0.091, *SE* = 0.020, *p* < 0.001) compared with those with earlier AoAs (Fig. 6). There were no main effects of ISI, nor any interactions between any of the variables (Table 6). This result follows our hypothesis that later AoA would result in more errors overall and again shows that novel pseudosigns are more challenging than known real signs.

#### Parameter accuracy

Participants’ repetition errors were coded by parameter: handshape, location, movement, or orientation. In some trials, participants made an error in more than one parameter (e.g., a handshape error and a location error) in the same lexical repetition. When this occurred, each parameter error was counted individually towards each parameter in the following parameter error analyses.

These analyses use the percentage of errors (number of errors for each parameter out of the total number of errors) as opposed to the error rate (number of errors in each parameter out of the total number of trials). Because the above results showed that the error rate increased with AoA, using the percentage of errors allows us to investigate the pattern of parameter errors that might be hidden by overall higher error rates for late AoA signers. We also analyzed the error data separately for real signs and pseudosigns. Recall our hypothesis was that late AoA signers would have fewer handshape errors for real signs compared with early AoA signers and that the parameter errors for pseudosigns would show whether this was due to an inability to produce phonological forms or due to a difference in how the forms are processed when paired with meaning.

##### Real signs

**Fig. 7:**
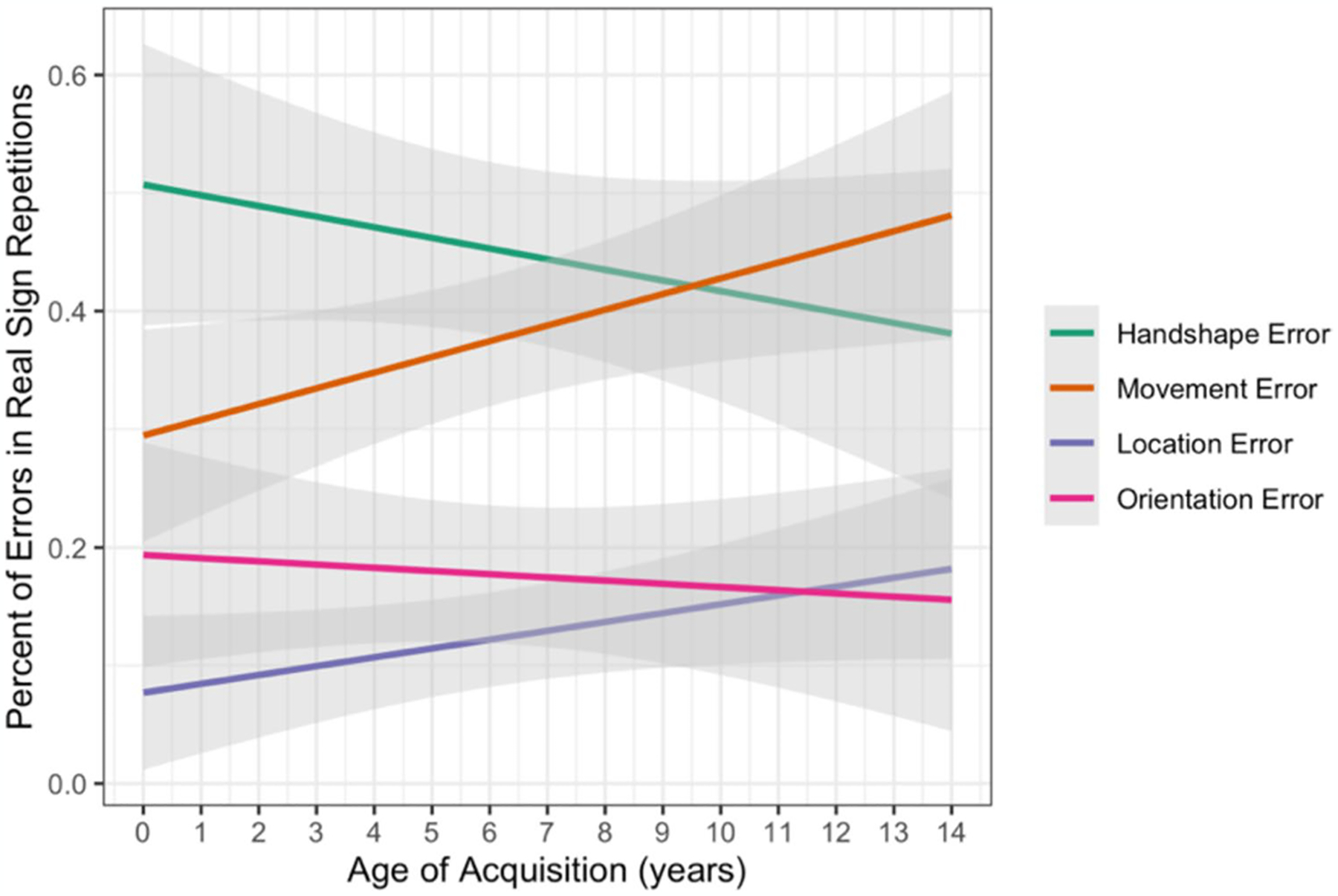
Percentage of errors in real sign repetitions by parameter and AoA with 95% confidence intervals.

We used linear regression modeling (lm) to test the fixed effects of parameter (handshape, movement, location, orientation) and AoA, along with interactions between the effects, on the continuous outcome variable of percentage of errors for each participant on real sign trials. Treatment coding was used with handshape as the reference level for parameter. There was an interaction between AoA and percentage of movement errors (β = 0.022, *SE* = 0.008, *p* = 0.009) such that participants with later AoAs had a higher percentage of movement errors compared with those with earlier AoAs. As predicted, the percentage of handshape errors decreased with AoA and the percentage of movement errors increased with AoA (see Fig. 7). While not significant, the percentage of location errors also trended towards increasing with AoA while the percentage of orientation errors remained relatively stable across AoA (Fig. 7; Table 7). Thus, participants with later AoAs showed a different pattern of parameter errors when repeating real signs (i.e., more errors in movement than the other parameters) compared with those with earlier AoAs (i.e., more errors in handshape than the other parameters).

##### Pseudosigns

**Fig. 8:**
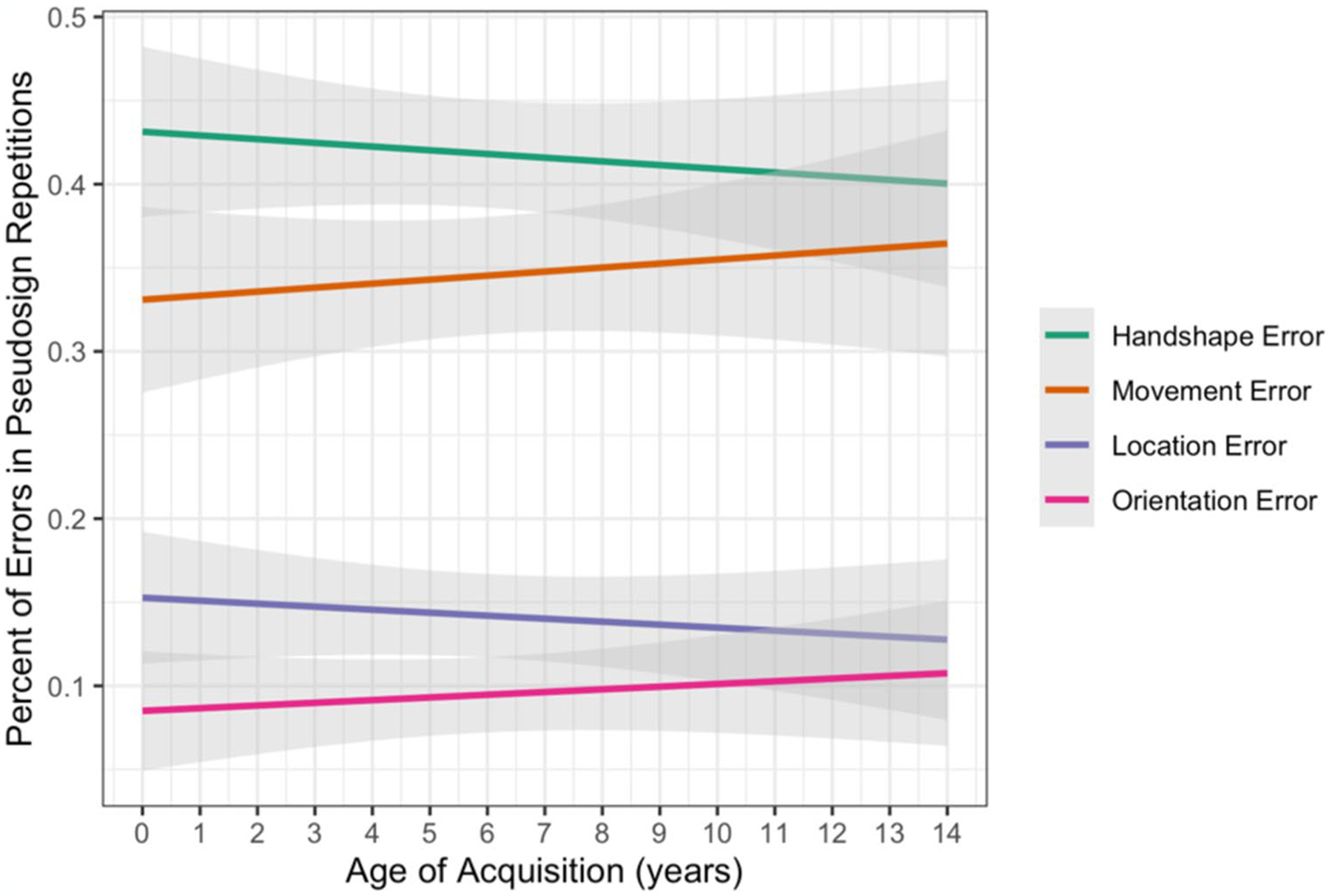
Percentage of errors in pseudosign repetitions by parameter and AoA with 95% confidence intervals.

We used linear regression modeling (lm) to test the fixed effects of parameter (handshape, movement, location, orientation) and AoA, along with interactions between the effects, on the continuous outcome variable of percentage of errors for each participant on pseudosign trials. Treatment coding was used with handshape as the reference level for parameter. There were no interactions between AoA and any of the parameters (Table 8). This indicates that the participants, regardless of AoA, had the same pattern of parameter errors (i.e., more errors in handshape than the other parameters) when repeating possible pseudosigns (see Fig. 8).

### Lexical repetition summary

We analyzed lexical repetition response time, production time, and accuracy as a function of AoA for real signs and possible pseudosigns. The pseudosigns elicited longer response times and higher error rates regardless of AoA. Likewise, production times were longer for the pseudosigns regardless of AoA, indicating participants were similarly affected by meaningless forms. However, AoA did affect repetition accuracy with later AoA resulting in more phonological errors overall and more movement than handshape errors. This pattern was the opposite of the one seen for participants with earlier AoAs, but only for real signs. The pattern of parameter errors for pseudosign repetitions was unaffected by AoA with more errors occurring in handshape than movement regardless of AoA. Altogether, the lexical repetition results indicate that AoA affected phonological processing of the parameters in real signs despite not affecting the ability to produce phonological forms in pseudosign processing.

## General discussion

Using both lexical decision and lexical repetition tasks, we investigated AoA effects on phonological processing in ASL. The lexical decision results showed that participants were sensitive to ASL phonological structure regardless of AoA. However, the lexical repetition task showed AoA to affect both the overall accuracy and type of phonological errors the participants produced, though only for real ASL signs and not pseudosigns. These results corroborate previous research and extend it in ways that provide more insight into AoA effects on phonology processing in ASL.

The lexical decision task required knowledge of ASL phonology and lexicality, and the results showed that the participants, regardless of AoA, indeed had this knowledge. Participants were slower to respond to pseudosigns compared with real signs and slower still to respond to phonotactically impossible pseudosigns than phonotactically possible ones. In addition, participants were more accurate in their lexical decisions when determining that a phonotactically impossible pseudosign was not a real ASL sign compared with the phonotactically possible pseudosigns. While these pseudosign results may be unsurprising, it is important to recognize these effects were independent of AoA, indicating that the participants with later AoAs were sensitive to the phonological structure of ASL.

We then investigated AoA effects on how that structure is processed through a lexical repetition task. Performance on the real sign repetitions followed our hypothesis that AoA would affect the overall error rate and the parameter errors. Specifically, those with earlier AoAs had a higher percentage of handshape errors than all the other parameters independent of whether the phonological form had meaning, as expected. In contrast, those with later AoAs had a higher percentage of movement errors when repeating real signs, resulting in an altered pattern of parameter errors for real signs. Performance on the pseudosign repetitions, on the other hand, showed no effect of AoA. Along with the evidence from the lexical decision task that late AoA signers are sensitive to phonological structure in ASL, these findings suggest that later AoA does not inhibit the ability to utilize and produce ASL phonology in pseudosigns but does alter how the parameters are processed when phonological forms have meaning.

Research has shown that late AoA signers rely more heavily on meaning and world knowledge over abstract linguistic cues like word order (Cheng & Mayberry, 2021; Malaia et al., 2020), and the effect of lexicality seen in the current study further supports this. It is possible that processing of meaning in the real signs took precedence over phonological processing for the late AoA signers, leading to a different pattern of parameter errors for real signs. In contrast, phonological processing was not inhibited by meaning in the pseudosigns and thus the parameter errors for late AoA signers resembled that of early AoA signers. This then leads to the question of why the parameters are processed differently in real signs by late AoA signers.

We found that handshape errors decreased with AoA (Fig. 7), which fits well with the previous research showing that late AoA signers over rely on handshape when monitoring signs (Morford & Carlson, 2011) and when making similarity judgments (Hildebrandt & Corina, 2002). Late AoA signers are also less categorical than early AoA signers in their perception of handshape (Morford et al., 2008) and reach high levels of production accuracy for handshapes (Morford, 2000). Together these findings indicate that handshape is especially salient when ASL is acquired after early childhood.

This finding for handshape may be surprising given that handshape is the most challenging parameter for child signers to accurately produce (Bonvillian & Siedlecki, 2000; Conlin et al., 2000; Siedlecki & Bonvillian, 1993) and the adults in this study with earlier AoAs made the most errors in handshape. A predominance of handshape errors has also been observed in hearing L2 signers (Ortega & Morgan, 2010, 2015). However, handshape is identified early in sign recognition (Clark & Grosjean, 1982; Emmorey & Corina, 1990; Grosjean, 1981; Morford & Carlson, 2011), making it accessible early in sign processing. Handshape may also be more perceptually stable and more iconically motivated than movement, leading it to be a more dependable lexical recognition cue for late AoA signers (Morford & Carlson, 2011; see also work looking at the relationship between handshape and form-meaning mapping, e.g., Occhino et al., 2017, 2020). Even research with homesigners, individuals born profoundly deaf who receive no sign input and consequently create their own gesture system to communicate with their hearing families, finds that the handshapes they use to signal meaning are more similar to those of natural sign languages than to the gestures of hearing speakers (Brentari et al., 2012). This suggests that handshape processing may be less susceptible to effects of insufficient language input in early childhood.

On the other hand, the percentage of movement errors increased with AoA (Fig. 7), prompting the question of why movement may be processed differently for late AoA signers. Unlike handshape or location, the movement parameter carries morphological information with changes in movement signaling morphological and aspectual marking in ASL.^6^ Moreover, movement is contrastive at multiple levels of linguistic structure. Meaningful morphological changes in movement, required for both inflectional and derivational morphology, differ from lexical changes in movement and also from classifier or depiction movements. Late AoA signers may have difficulty with movement because they have not separately learned and incorporated these different kinds of movement into their morphophonological system. A related possibility, as noted above, is that handshape is a more stable and reliable feature than the movement parameter when sign exposure occurs after early childhood, leading to less attention to meaningful contrasts in movement which are more dynamic and less stable.

Movement is also unique in that it is the parameter identified last in sign recognition gating experiments after location and handshape (Clark & Grosjean, 1982; Emmorey & Corina, 1990; Grosjean, 1981; Morford & Carlson, 2011) and is the least often recalled parameter in “tip-of-the-finger” experiences (Thompson et al., 2005). It is necessary to identify or recall movement to fully access a sign, but perhaps a delay in access to movement more dramatically affects signers with later AoA. When performing gating experiments, signers with later AoAs take longer to identify signs in the early stages of lexical access (Emmorey & Corina, 1990), which could be caused by a delay in access to movement.

An intriguing and related observation is that emerging sign languages differ from older sign languages regarding movement in the early stages of development. In older sign languages like ASL, movement is required in every sign, even if it is physically and phonetically possible to not have a movement within the sign (Brentari, 1990; Stack, 1988; Wilbur, 1987). Evidence from Al-Sayyid Bedouin Sign Language (ABSL), though, shows that in the first generations of its development movement was not required in signs and was frequently left out when it was physically possible to not have a movement (Israel, 2009; Sandler et al., 2011). There are many differences between community development of a new sign language and the acquisition of an older language at a later AoA. However, the finding that ABSL did not immediately have a requirement that every sign have a movement coupled with the present result that late AoA signers may have difficulty with movement could indicate that segmenting the dynamic signal of movement for linguistic meaning is an abstract phonemic skill best acquired in early childhood with regular sign input. This is supported by the finding that attention to and categorization of movement is affected by language experience (Poizner, 1981, 1983; Wilson, 2001). Thus, signers with later AoAs may be at a disadvantage for processing movement in real signs while being at an advantage for processing handshape due to attentional resources directed at handshape and not movement.

More research is needed to better understand why late AoA signers may be able to utilize phonology similarly to early AoA signers but ultimately still process the phonological parameters differently for real signs. Further work could more deeply investigate the differences seen here between handshape and movement, including looking more closely at forms within the parameters and not just at the parameter level. It is possible that certain kinds of movements (e.g., path vs. internal) or certain kinds of handshapes (e.g., marked vs. unmarked) are treated differently by late AoA signers. Additionally, sign complexity may be an important factor to investigate as it has also been shown to affect repetition accuracy for signing children (Gu et al., 2022). Importantly, these findings need to be replicated with different stimuli and in different tasks. While the stimuli in this study were created quite similarly to those in many other sign language studies, it is possible that the ways in which the pseudosigns differed from the real signs could have affected the results. Future work could utilize different kinds of pseudosigns to test the robustness of the lack of effect on pseudosign repetition errors. Other kinds of production tasks, including different kinds of repetition as well as naturalistic production, are needed to test the robustness of the effects seen in the real sign repetitions here. These kinds of more detailed investigations can better inform theoretical models and frameworks concerned with phonology. Further work can also provide more insight into additional factors like working memory, attentional control, and individual differences that were not explored here.

### Conclusion

The aim of this study was to investigate the effects of AoA on phonological parameter production in ASL. In a lexical decision task, participants across AoA showed sensitivity to ASL phonological structure and lexicality, allowing us to then investigate how participants produce that structure in a lexical repetition task with both real signs and possible pseudosigns in ASL. Participants had similar error rates and patterns of parameter errors for pseudosign repetitions regardless of AoA, suggesting that those with later AoAs were able to utilize and produce ASL phonology like those with earlier AoAs. Despite this, AoA effects were present in the real sign repetitions. Those with later AoAs had more overall phonological errors and a different pattern of parameter errors for real ASL signs, with movement errors increasing and handshape errors decreasing with AoA. Ultimately, this study provides evidence that later AoA does not inhibit the ability to produce ASL phonology but alters production of phonological parameters when the processing of meaning and phonology are integrated.

## Supplementary Material

Supplemental Materials

## Figures and Tables

**Table 1:** Distribution of participants across age of acquisition of ASL as a first language

Age of acquisition	Number of participants
0 years old	18
2 years old	2
3 years old	2
4 years old	2
5 years old	5
6 years old	7
7 years old	5
8 years old	1
9 years old	3
10 years old	2
11 years old	2
12 years old	4
13 years old	8
14 years old	2

**Table 2:** Statistical model output for lexical decision response time

Variable	Estimate	Standard error	*df*	*t* value	Pr(>|t|)
(Intercept)	1705.704	80.165	62.951	21.277	< 0.0000000000000002***
ISI [S.300]	− 24.114	19.178	432.348	1.257	0.209
AoA	19.358	10.556	58.730	1.834	0.07175
Condition [S.Possible]	207.709	33.728	457.149	6.158	0.00000000161***
Condition [S.Impossible]	383.483	33.260	434.208	11.530	< 0.0000000000000002***
ISI [S.300]: AoA	− 0.929	1.601	14,497.923	− 0.580	0.56194
ISI [S.300]:Condition[S.Possible]	− 31.706	33.696	455.463	− 0.941	0.34723
ISI [S.300]:Condition[S.Impossible]	14.361	33.245	433.438	0.432	0.66597
AoA: Condition[S.Possible]	− 1.590	4.347	14,513.399	− 0.366	0.71452
AoA: Condition[S.Impossible]	− 7.120	4.119	14,490.204	− 1.728	0.08392
ISI [S.300]:AoA: Condition [S.Possible]	− 4.999	2.869	14,503.183	− 1.743	0.08141
ISI [S.300]: AoA: Condition [S.Impossible]	− 1.099	2.7789	14,491.555	− 0.396	0.69225

Response Time ~ Interstimulus Interval (ISI) × Age of Acquisition (AoA) × Condition + (1|Subject) + (1|Trial).

**Table 3:** Statistical model output for lexical decision accuracy

Variable	Estimate	Standard error	*z* value	Pr(>|t|)
(Intercept)	− 2.290	0.159	− 15.685	< 0.0000000000000002***
ISI[S.300]	0.117	0.082	1.430	0.152641
AoA	0.036	0.019	1.948	0.051375
Condition[S.Possible]	0.783	0.134	5.850	0.00000000491***
Condition[S.Impossible]	0.080	0.139	0.577	0.563609
ISI[S.300]:AoA	− 0.005	0.006	− 0.822	0.411029
ISI[S.300]:Condition[S.Possible]	− 0.071	0.134	− 0.527	0.598027
ISI[S.300]:Condition[S.Impossible]	0.098	0.139	0.708	0.478635
AoA: Condition[S.Possible]	− 0.016	0.009	− 1.768	0.077055
AoA: Condition[S.Impossible]	− 0.010	0.010	− 0.958	0.338305
ISI[S.300]:AoA: Condition[S.Possible]	0.004	0.009	0.486	0.627293
ISI[S.300]:AoA: Condition[S.Impossible]	0.001	0.010	0.065	0.947992

Error Rate ~ Interstimulus Interval (ISI) × Age of Acquisition (AoA) × Condition + (1|Subject) + (1|Trial).

**Table 4:** Statistical model output for lexical repetition response time

Variable	Estimate	Standard error	*df*	*t* value	Pr(>|t|)
(Intercept)	1286.036	27.423	98.430	46.896	< 0.0000000000000002***
ISI[S.300]	− 15.073	13.102	213.238	− 1.150	0.2512
AoA	3.133	3.145	61.406	0.996	0.3230
Condition[S.Possible]	124.179	19.118	215.055	6.495	0.000000000566***
ISI[S.300]:AoA	− 0.351	0.326	11,504.472	1.075	0.2822
ISI[S.300]: Condition[S.Possible]	− 35.859	19.117	215.019	1.876	0.0620
AoA: Condition[S.Possible]	− 0.7462	0.507	11,507.434	− 1.472	0.1412
ISI[S.300]:AoA: Condition[S.Possible]	0.6121	0.5061	11,506.735	1.210	0.2265

Response Time ~ Interstimulus Interval (ISI) × Age of Acquisition (AoA) × Condition + (1|Subject) + (1|Trial).

**Table 5:** Statistical model output for lexical repetition production time

Variable	Estimate	Standard error	*df*	*t* value	Pr(>|t|)
(Intercept)	1401.256	45.002	65.743	31.137	< 0.0000000000000002***
ISI[S.300]	− 34.802	18.964	239.089	− 1.835	0.067718
AoA	5.729	5.739	61.238	0.998	0.322107
Condition[S.Possible]	78.617	13.359	248.619	5.885	0.0000000128***
ISI[S.300]:AoA	0.283	0.444	11,449.449	0.637	0.524
ISI[S.300]: Condition[S.Possible]	− 17.372	13.357	248.463	− 1.301	0.194593
AoA: Condition[S.Possible]	− 0.527	0.690	11,457.826	− 0.764	0.445153
ISI[S.300]:AoA: Condition[S.Possible]	− 0.552	0.689	11,457.359	− 0.801	0.423065

Production Time ~ Interstimulus Interval (ISI) × Age of Acquisition (AoA) × Condition + (1|Subject) + (1|Trial).

**Table 6:** Statistical model output for lexical repetition accuracy

Variable	Estimate	Standard error	*z* value	Pr(>|t|)
(Intercept)	− 3.980	0.195	− 20.409	< 0.0000000000000002***
ISI[S.300]	− 0.037	0.146	− 0.253	0.8001
AoA	0.091	0.020	4.429	0.00000945***
Condition[S.Possible]	2.041	0.181	11.250	< 0.0000000000000002***
ISI[S.300]:AoA	0.013	0.012	1.028	0.3040
ISI[S.300]: Condition[S.Possible]	0.019	0.179	0.106	0.9157
AoA: Condition[S.Possible]	− 0.033	0.014	− 2.361	0.0182
ISI[S.300]:AoA: Condition[S.Possible]	− 0.009	0.014	− 0.624	0.5325

Error Rate ~ Interstimulus Interval (ISI) × Age of Acquisition (AoA) × Condition + (1|Subject) + (1|Trial).

**Table 7:** Statistical model output for lexical repetition real sign parameter errors

Variable	Estimate	Standard error	*t* value	Pr(>|t|)
(Intercept)	0.507	0.047	10.766	< 0.0000000000000002***
AoA	− 0.009	0.006	− 1.500	0.13484
Location	− 0.430	0.067	− 6.458	0.000000000601***
Movement	− 0.213	0.067	− 3.191	0.00161**
Orientation	− 0.313	0.067	− 4.704	0.000004339275***
AoA:Location	0.017	0.008	1.943	0.05320
AoA:Movement	0.022	0.008	2.629	0.00913**
AoA:Orientation	0.006	0.008	0.741	0.45959

Percent Error ~ Parameter × Age of Acquisition (AoA); handshape as reference level.

**Table 8:** Statistical model output for lexical repetition pseudosign parameter errors

Variable	Estimate	Standard error	*z* value	Pr(>|t|)
(Intercept)	0.431	0.023	18.691	< 0.0000000000000002***
AoA	− 0.002	0.003	− 0.740	0.45979
Location	− 0.279	0.033	− 8.537	0.00000000000000149***
Movement	− 0.100	0.033	− 3.077	0.00233**
Orientation	− 0.346	0.033	− 10.611	< 0.0000000000000002***
AoA:Location	0.001	0.004	0.100	0.92078
AoA:Movement	0.005	0.004	1.091	0.27636
AoA:Orientation	0.004	0.004	0.904	0.36712

Percent Error ~ Parameter × Age of Acquisition (AoA); handshape as reference level.

## Data Availability

Videos of all stimuli are available online (https://osf.io/n23wh/). The datasets and other materials generated during and/or analyzed during the current study are available from the corresponding author upon reasonable request.

## Footnotes

1 Here, we distinguish late AoA signers from second language signers as we are discussing deaf individuals who did not have sufficient language exposure in early childhood for eventual acquisition of ASL to be considered a second language. These deaf signers are also referred to as late first language signers (e.g., Cheng & Mayberry, 2021), late learners (e.g., Hildebrandt & Corina, 2002), or nonnative signers (e.g., Hall et al., 2012) across sign language research.

2 Nonmanual markers are considered a fifth parameter (Hill et al., 2019), though due to their sparse participation in meaningful contrasts and greater participation at the morphological and syntactic levels, they will not be discussed in this paper.

3 Links to online videos of signs are courtesy of ASL-LEX 2.0, a publicly accessible database of ASL signs (Sehyr et al., 2021). English glosses for ASL signs are given in all caps. However, English glosses do not always indicate a direct translation and do not necessarily represent the limit of meaning for a given sign.

4 These data were collected in 1999–2003 at McGill University before there was any ASL corpus data available. The participants were born and educated in Quebec and Ontario in the 1970s, when clinical and educational services for deaf and hard of hearing infants and children used an auditory–oral approach that eschewed any exposure to sign languages or gesture.

5 As discussed in the General Methods, recruiting deaf participants equally across every AoA can be difficult. Because of this, some AoAs have fewer participants than others (Table 1) and in some figures some AoAs with fewer participants may visually appear to be outliers. It is important to note that while some of these AoAs with fewer participants appear visually to be outliers, others also with fewer participants do not, meaning it is not possible to determine which are outliers and which are not. Thus, we did not exclude any participants from analyses based on appearing to be outliers in figures as it is our view that more data is more helpful here.

6 For example, a change in movement in the ASL sign LOOK-AT can convey, for example, “look at for a long time,” “look at incessantly,” “look at habitually” (Sandler & Lillo-Martin, 2006).

## Appendix 1. Stimulus items in the sign pair trials

Videos of all stimuli are available online (https://osf.io/n23wh/). The English glosses for the signs correspond to those used in Costello (1994).

**Table 9:** Stimuli in the unrelated real sign condition

Prime (real sign)	Target (real sign)	Interstimulus interval (ms)	OSF link
ACT	FRUIT	300	https://osf.io/u5x3a
HAIRDRYER	BIRTH	300	https://osf.io/e8x6r
BAKE	ARMY	300	https://osf.io/fkwgd
HORSE	ABOUT	300	https://osf.io/ew749
CITY	NEPHEW	300	https://osf.io/2e7gb
EXPERIENCE	APPEAR	300	https://osf.io/pkvba
BEER	INTERNET	300	https://osf.io/h8fkv
COMB	MOCK	300	https://osf.io/k4swj
CRUEL	ASK	300	https://osf.io/c47qr
GREECE	MEASURE	300	https://osf.io/xd7j6
LEAVE	ENGAGEMENT	300	https://osf.io/3sfvx
FARM	REASON	300	https://osf.io/uqtzw
KNOB	DRUNK	300	https://osf.io/ngm8b
CLERGY	ISLAND	300	https://osf.io/smbkx
HOTDOG	PARENTS	300	https://osf.io/jb5m3
HUNT	LIGHT (weight)	300	https://osf.io/b8jw4
RUDE	MISS/GONE	300	https://osf.io/jhpzg
GALLAUDET	COOKIE	300	https://osf.io/fu7mj
FOOD	RADIO	300	https://osf.io/fjvp2
KNEEL	HEALTH	300	https://osf.io/nk5us
LECTURE	SHIRT	300	https://osf.io/q4azc
LONELY	APPOINTMENT	300	https://osf.io/2n9kr
TOILET	BASKETBALL	300	https://osf.io/a83fs
MAGAZINE	BECAUSE	300	https://osf.io/s5qe9
FRENCH-FRIES	CHARACTER	300	https://osf.io/xfjr3
MONKEY	CANDY	300	https://osf.io/tmpfb
MUSTACHE	PUNISH	300	https://osf.io/jqrc8
DELICIOUS	YOURSELF	300	https://osf.io/esmxy
MOUTH	NURSE	300	https://osf.io/t74ju
BOWL	MAN	300	https://osf.io/h5q8m
PEACE	COUGH	300	https://osf.io/h4vrm
CAPTION	INSECT	300	https://osf.io/tydz7
PITY	CAMERA	300	https://osf.io/uczv4
POISON	TALL	300	https://osf.io/z48cw
SHY	PRICE	300	https://osf.io/xefwq
MONDAY	BUTTERFLY	300	https://osf.io/rnpzs
CENTER	FAULT	1,000	https://osf.io/aqgup
MIND	RABBIT	1,000	https://osf.io/wv6jr
WRISTWATCH	TO SMOKE	1,000	https://osf.io/3tx6h
MANNERS	THURSDAY	1,000	https://osf.io/d6qar
RESPONSIBLE	PRETTY	1,000	https://osf.io/ebnwm
HAMBURGER	ISRAEL	1,000	https://osf.io/e7fwu
FRIENDLY	THIRST	1,000	https://osf.io/tv5d4
HAMMER	CIGARETTE	1,000	https://osf.io/yp5mc
FAST	STUBBORN	1,000	https://osf.io/m4nh6
LAUGH	SHOPPING	1,000	https://osf.io/h2m7c
SENTENCE	CANADA	1,000	https://osf.io/2dx4z
BOY	SALAD	1,000	https://osf.io/ufs8t
DOESN’T-MATTER	PUZZLED	1,000	https://osf.io/hu3m2
WHAT-FOR	SOME	1,000	https://osf.io/ux9j3
GRASS	WONDER	1,000	https://osf.io/asy9k
BASEBALL	KEY	1,000	https://osf.io/uqk7n
CUP	DRAMA	1,000	https://osf.io/twe34
HONOR	CHECK	1,000	https://osf.io/4bcpw
HAIR	FREEWAY	1,000	https://osf.io/9fkgd
STRESS	CERTIFICATE	1,000	https://osf.io/fuavd
SUSPECT	CONGRATULATIONS	1,000	https://osf.io/skex5
MOTHER	SUNSET	1,000	https://osf.io/q52ps
THING	UNIVERSITY	1,000	https://osf.io/hjeab
SURPRISE	BRIDGE	1,000	https://osf.io/rdq9v
TELEVISION	BODY	1,000	https://osf.io/azphc
THIEF	RESEARCH	1,000	https://osf.io/mbpzw
TIRED	HONEST	1,000	https://osf.io/rkuqa
COLD	STRICT	1,000	https://osf.io/aw6v8
FRONT	TYPE/KIND	1,000	https://osf.io/s3ad2
BLOOD	UMBRELLA	1,000	https://osf.io/acuzy
SNOB	BREAKDOWN	1,000	https://osf.io/crgjt
CHALLENGE	MORNING	1,000	https://osf.io/8dqkv
POWER	WAIT	1,000	https://osf.io/fx9r8
WASH MACHINE	HIGH SCHOOL	1,000	https://osf.io/6ghkt
SALT	WEAR	1,000	https://osf.io/8e57c
WEEK	STOMACH	1,000	https://osf.io/r6d8b

**Table 10:** Stimuli in the possible pseudosign condition

Prime (real sign)	Target (pseudosign)	Parameter changed in target	Interstimulus interval (ms)	OSF link
BANANA	TELL	Handshape	300	https://osf.io/a5ndh
SHORTS	TEAR (cry)	Handshape	300	https://osf.io/xkuqt
HOUR	PULL	Handshape	300	https://osf.io/y6p47
SILLY	ARRIVE	Handshape	300	https://osf.io/65waf
BOOK	PROUD	Handshape	300	https://osf.io/4ftqr
FOOTBALL	MAD	Handshape	300	https://osf.io/fuqhs
LETTER	BLAME	Handshape	300	https://osf.io/dt4ez
DIVORCE	BOAT	Handshape	300	https://osf.io/5hnkc
MISUNDERSTAND	VOLUNTEER	Handshape	300	https://osf.io/u6n2p
QUIET	STOP	Handshape	300	https://osf.io/yq9hd
WASTE	DOWN	Handshape	300	https://osf.io/zeyjd
REQUIRE	FORCE	Handshape	300	https://osf.io/7xd98
SLOW	FLUNK (fail)	Handshape	1,000	https://osf.io/z938x
SPAGHETTI	LOUSY	Handshape	1,000	https://osf.io/pkevz
SUBTRACT	ON	Handshape	1,000	https://osf.io/tu75s
WORLD	THINK	Handshape	1,000	https://osf.io/4rew3
OTHER	SISTER	Handshape	1,000	https://osf.io/snbjp
SAME	SNOW	Handshape	1,000	https://osf.io/pbfm8
HUNGRY	SCIENCE	Handshape	1,000	https://osf.io/ednw4
ICE CREAM	IMPROVE	Handshape	1,000	https://osf.io/9284b
UNDERSTAND	PLANE	Handshape	1,000	https://osf.io/je9pc
WHO	SPELL	Handshape	1,000	https://osf.io/eumy9
STRANGE	REST	Handshape	1,000	https://osf.io/ezr8q
GOVERNMENT	DIZZY	Handshape	1,000	https://osf.io/x7n4k
BABY	COLD (sick)	Location	300	https://osf.io/6mkr2
BEHIND	STUCK	Location	300	https://osf.io/9gnak
BOX	STUPID	Location	300	https://osf.io/2juyq
BREAD	ONE	Location	300	https://osf.io/s6d2v
BROTHER	GARBAGE	Location	300	https://osf.io/5by7a
CHILDREN	TOMORROW	Location	300	https://osf.io/7f2qx
AMERICA	DRY	Location	300	https://osf.io/yt7xz
SCISSORS	ONCE	Location	300	https://osf.io/vhe5c
GLASSES	RESIST	Location	300	https://osf.io/mrnwg
HAVE	SHAME	Location	300	https://osf.io/t5pvs
MUCH	CLEAN	Location	300	https://osf.io/4ravx
PIPE	FIVE	Location	300	https://osf.io/ch9md
VICTIM	MEMBER	Location	1,000	https://osf.io/p2bfy
HARD	WRONG	Location	1,000	https://osf.io/j2r6k
DRINK	DUTY	Location	1,000	https://osf.io/wb9yv
EXPENSIVE	DIRTY	Location	1,000	https://osf.io/ug4c3
DRAWER	NIECE	Location	1,000	https://osf.io/twv3x
JACKET	ELECTRICITY	Location	1,000	https://osf.io/vhc62
MIRROR	ADVICE	Location	1,000	https://osf.io/tykmc
ORANGE	CONTACT	Location	1,000	https://osf.io/ywvk6
BORROW	WISE	Location	1,000	https://osf.io/7fd2j
TURTLE	SPANISH	Location	1,000	https://osf.io/qe4g7
WEDNESDAY	ANALYZE	Location	1,000	https://osf.io/dc8tg
EARRING	CARELESS	Location	1,000	https://osf.io/dxqag
CAPTAIN	COPY	Movement	300	https://osf.io/4pnqy
BEARD	WHITE	Movement	300	https://osf.io/9vyg8
BREAK	SKILL	Movement	300	https://osf.io/5uqna
DECIDE	FROM	Movement	300	https://osf.io/chgvw
BAD	LIBRARY	Movement	300	https://osf.io/3tbr4
DREAM	SIT	Movement	300	https://osf.io/a2zrs
DROP	HIT	Movement	300	https://osf.io/dzpkx
FINISH	LEAD	Movement	300	https://osf.io/k7uza
BLOCKHEAD	MUST	Movement	300	https://osf.io/z8y5j
GOOD	BED	Movement	300	https://osf.io/7zh5u
GRANDFATHER	RUN-AWAY	Movement	300	https://osf.io/8mup7
GROW	SEE	Movement	300	https://osf.io/mq2dg
ANGRY	LAZY	Movement	1,000	https://osf.io/4hxzy
HEADACHE	NOT	Movement	1,000	https://osf.io/hfg4r
DEEP	JAIL	Movement	1,000	https://osf.io/8uq7d
CABINET	DISCUSS	Movement	1,000	https://osf.io/anm7u
LIPSTICK	BITE	Movement	1,000	https://osf.io/zh345
COLLEGE	LION	Movement	1,000	https://osf.io/znh4x
DISAGREE	MELT	Movement	1,000	https://osf.io/4dsy9
FLOWER	SEPARATE	Movement	1,000	https://osf.io/9f7ds
GREEN	STAY	Movement	1,000	https://osf.io/q5pk9
NEW YORK	UNCLE	Movement	1,000	https://osf.io/tg75n
MILK	BROOM	Movement	1,000	https://osf.io/zbyfe
COUSIN	NUTS	Movement	1,000	https://osf.io/dvz5e

**Table 11:** Stimuli in the impossible pseudosign condition

Prime sign (real)	Target sign (pseudo)	Parameter changed in target sign	Interstimulus interval (ms)	OSF link
AFRAID	EAST	Handshape	300	https://osf.io/eas64
FAVORITE	NAME	Handshape	300	https://osf.io/4wqbv
CANCEL	DUMB	Handshape	300	https://osf.io/w4p5r
FRECKLES	WITH	Handshape	300	https://osf.io/7xeds
PILE	BOWLING	Handshape	300	https://osf.io/5a6bv
FIRE	MONTH	Handshape	300	https://osf.io/scnw3
POTATO	SIGN	Handshape	300	https://osf.io/d8svg
ORAL	NORTH	Handshape	300	https://osf.io/3nsmb
NOTHING	OPEN-WINDOW	Handshape	300	https://osf.io/ctda8
ENGLAND	NOSE	Handshape	300	https://osf.io/4qzuy
IMAGINE	CAT	Handshape	300	https://osf.io/v98mq
HOSPITAL	UP	Handshape	300	https://osf.io/g452q
THAT	HEAVY	Handshape	1,000	https://osf.io/e3z7m
RELATIONSHIP	FOOL	Handshape	1,000	https://osf.io/ez4ay
FRIDAY	ONE-CENT	Handshape	1,000	https://osf.io/e4mr9
MOON	GERMAN	Handshape	1,000	https://osf.io/njwth
SHOWER	POSTPONE	Handshape	1,000	https://osf.io/hck6b
DAY	OFFER	Handshape	1,000	https://osf.io/kmwxv
LOSE-GAME	YELLOW	Handshape	1,000	https://osf.io/2a6cr
BAGGAGE	FUTURE	Handshape	1,000	https://osf.io/kw3g9
INVITE	FUNNY	Handshape	1,000	https://osf.io/ry46q
PROTECT	SELL	Handshape	1,000	https://osf.io/6cgp5
TRAFFIC	TAKE	Handshape	1,000	https://osf.io/46a3c
TALK	THANKS	Handshape	1,000	https://osf.io/x3b4p
WEAK	ALL	Location	300	https://osf.io/rp83m
PIG	TICKET	Location	300	https://osf.io/zc9w3
BRAVE	WOOD	Location	300	https://osf.io/d8rmn
70	KEEP	Location	300	https://osf.io/jmcwh
UGLY	YOUNG	Location	300	https://osf.io/vu68e
EQUAL	TREE	Location	300	https://osf.io/qs2fp
BUTTER	BROOCH (pin)	Location	300	https://osf.io/xu92a
VOICE	WINDOW	Location	300	https://osf.io/e5dzj
MAKE	TEASE	Location	300	https://osf.io/4zpj8
WARM	BELIEVE	Location	300	https://osf.io/zu2fv
BLUE	YESTERDAY	Location	300	https://osf.io/9dyf8
WARN	ADDRESS	Location	300	https://osf.io/bvp43
SWALLOW	YES	Location	1,000	https://osf.io/dt6jv
AGREE	DENY	Location	1,000	https://osf.io/mh2vf
SON	TUESDAY	Location	1,000	https://osf.io/6ct5u
LOOK-FOR	HEAD	Location	1,000	https://osf.io/j3ach
KNOW	HURRY	Location	1,000	https://osf.io/rygak
KID	MIDDLE	Location	1,000	https://osf.io/2qz8h
AFTER	EGG	Location	1,000	https://osf.io/67tah
STAMP	GUESS	Location	1,000	https://osf.io/3f7mc
VALUES	DICTIONARY	Location	1,000	https://osf.io/h2ja4
SPEAK	WASHINGTON	Location	1,000	https://osf.io/ezyq6
LIFE	GUILTY	Location	1,000	https://osf.io/3e6xb
SORRY	EARTH	Location	1,000	https://osf.io/g8qb4
DEBT	ANNIVERSARY	Movement	300	https://osf.io/wq7vp
GRANDMOTHER	BEAR	Movement	300	https://osf.io/rakjt
HEARING-AID	BICYCLE	Movement	300	https://osf.io/ha9tz
HEART	ARTICLE	Movement	300	https://osf.io/gvrpj
MULTIPLY	GIVE	Movement	300	https://osf.io/jprd8
POOR	INTELLIGENT	Movement	300	https://osf.io/ewgpd
HOME	BREATHE	Movement	300	https://osf.io/sc543
HEARING	ART	Movement	300	https://osf.io/qx4ju
CHAT	FIND	Movement	300	https://osf.io/5w2cb
KNIFE	SKIRT	Movement	300	https://osf.io/ugmfj
JEWISH	APPLAUD	Movement	300	https://osf.io/ub6ts
PARADE	GULLIBLE	Movement	300	https://osf.io/v2mkb
VACATION	DOCTOR	Movement	1,000	https://osf.io/yh4vn
ACCIDENT	GOAL	Movement	1,000	https://osf.io/a8yed
MACHINE	AUNT	Movement	1,000	https://osf.io/ub5fr
EGYPT	ALONE	Movement	1,000	https://osf.io/ngr5w
INDIAN	CHARGE	Movement	1,000	https://osf.io/cwhqd
AGE	DOOR	Movement	1,000	https://osf.io/cjrp7
EUROPE	ZERO	Movement	1,000	https://osf.io/rkhs6
ESTABLISH	ADMIT	Movement	1,000	https://osf.io/2hf6j
EMBARRASS	ANY	Movement	1,000	https://osf.io/h7f8s
GRADUATE	COMMITTEE	Movement	1,000	https://osf.io/5erbg
EXPLAIN	BACK	Movement	1,000	https://osf.io/xaebt
MOVIES	BLUNT	Movement	1,000	https://osf.io/hzrb2
