# Supplementary material for: Production of real signs but not pseudosigns affected by age of acquisition in American Sign Language
Source: Mem Cognit. 2025 Jan 22;53(5):1356–79. doi: 10.3758/s13421-024-01656-y (PMC12280091; doi:10.3758/s13421-024-01656-y)
Supplement: Supplementary file 1 — Supplementary file1 (DOCX 632 kb) [file 13421_2024_1656_MOESM1_ESM.docx]

**Supplemental Materials for:**

**Production of Real Signs but not Pseudosigns Affected by Age of Acquisition**

**in American Sign Language**

Shai Lynne Nielson & Rachel I. Mayberry

Department of Linguistics, University of California San Diego

**Supplemental Methods**

In these supplemental materials, we provide the analyses and discussion of the trials from the related real sign conditions. The related trials were a part of both the lexical decision and the lexical repetition tasks performed by all participants (described in the main text).

**Stimuli**

In addition to the 72 unrelated real sign pairs, there were 72 trials where real sign targets had one of three lexical-structural relationships to the real sign primes: phonological, semantic, and bilingual (English gloss rhyme).

Of these related trials, 24 formed a minimal phonological pair with the prime in ASL, sharing two of three parameters: handshape, location, and movement. For example, the ASL signs [BIRD](https://asl-lex.org/visualization/?sign=bird) and [NEWSPAPER](https://asl-lex.org/visualization/?sign=newspaper) share handshape and movement and differ only in location (i.e., mouth vs hand). Another 24 trials were semantically related to the prime in a superordinate category/exemplar fashion with the prime being a superordinate category, e.g. [FURNITURE](https://asl-lex.org/visualization/?sign=furniture), and the target being an exemplar, e.g., [CHAIR](https://asl-lex.org/visualization/?sign=chair). And another 24 trials of the ASL sign targets in the bilingual condition were related to the primes in terms of their English glosses’ rhyme, e.g., [FISH](https://asl-lex.org/visualization/?sign=fish) and [WISH](https://asl-lex.org/visualization/?sign=wish). For 20 of the 24 bilingual condition trials, the rhyme in English is apparent in the written forms (e.g., [LAST](https://asl-lex.org/visualization/?sign=last) and [PAST](https://asl-lex.org/visualization/?sign=past)). For 4 of the 24 bilingual condition trials, the rhyme was not apparent in the written forms (e.g., [NO](https://asl-lex.org/visualization/?sign=no) and [SEW](https://asl-lex.org/visualization/?sign=sew)).

The overall proportion of phonological, semantic, and bilingual priming items was intentionally kept low to increase the likelihood of obtaining priming effects and to discourage strategic responding (Neeley, 1991). Mean subjective frequency of the stimulus signs did not differ across the related conditions, nor did they vary as a function of the raters’ age of acquisition of ASL (Mayberry et al., 2014).

The stimulus creation, interstimulus intervals (ISI), trial structure, and experimental procedure for these conditions follow those described in the main text.

**Lexical Decision Analyses**

The lexical decision task is described in the main text. All models were fit using the lme4 (version 1.1-26; Bates et al., 2015), lmerTest (version 3.1-3; Kuznetsova et al., 2017), and car (version 3.1-2; Fox & Weisberg, 2019) packages in R (R Core Team, 2021). Sum coding was used for the two-level contrasts of ISI (300ms = +1, 1000ms = -1) and condition (phonologically related= +1, unrelated real signs = -1; semantically related = +1, unrelated real signs = -1; English gloss rhyme = +1, unrelated real signs = -1). All analyses were conducted with a threshold for significance of alpha = .01. When the variable of AoA was replaced by age at testing or years of experience in the following models, there was no effect of either variable.

**Response Time**

We used linear-mixed effects modeling (lmer) to test the fixed effects of age of acquisition, ISI, and trial condition (unrelated, phonological, semantic, bilingual), along with interactions between the effects, on the continuous outcome variable of response time in correct trials, with random intercepts for participants and trial. Subjective frequency^[[1]](#footnote-1)^ (Mayberry et al., 2014) and subjective iconicity^[[2]](#footnote-2)^ (Sehyr et al., 2021) of target ASL signs were included as covariates. As described above, sum coding was used for ISI and condition. Three participants did not have response times recorded due to equipment failure for their lexical decision trials, so they were excluded from response time analyses for the lexical decision task.

There were no main effects of age of acquisition, ISI, or any related conditions, nor any interactions between any of the variables (**Figure S1**). This suggests that decision response time was not modulated by the related conditions.

**Figure S1**


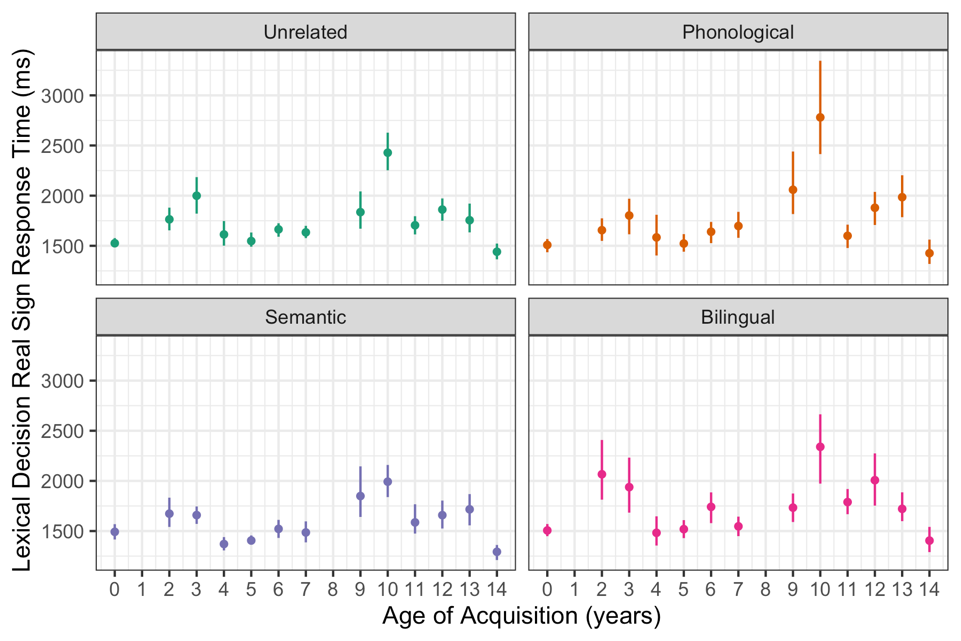


Lexical decision response time by trial condition and age of acquisition with 95% confidence intervals.

**Accuracy**

We used generalized binomial linear-mixed effects modeling (glmer) to test the fixed effects of age of acquisition, ISI, and trial condition (unrelated, phonological, semantic, bilingual), along with interactions between the effects, on the binary variable of lexical decision accuracy (0 = correct decision, 1 = incorrect decision), with random intercepts for participants and trial. Subjective frequency and subjective iconicity of target ASL signs were included as covariates. As described above, sum coding was used for ISI and condition.

There were no main effects of age of acquisition, ISI, or any related conditions, nor any interactions between any of the variables (**Figure S2**). This suggests that decision accuracy was not modulated by the related conditions.

**Figure S2**


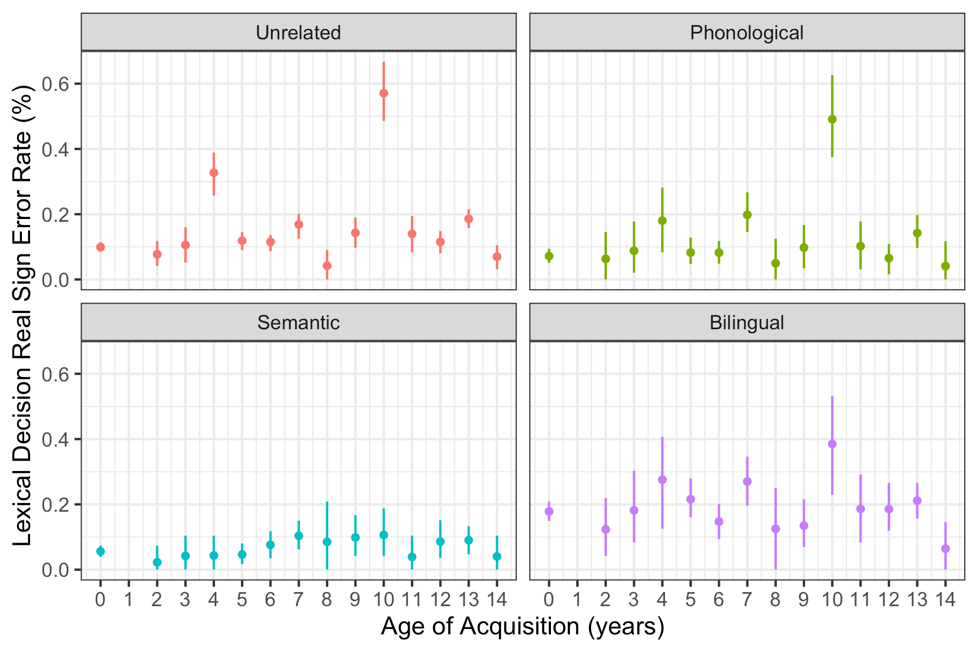


Lexical decision error rate by trial condition and age of acquisition with 95% confidence intervals.

**Lexical Repetition Analyses**

The lexical repetition task is described in the main text. All models were fit using the lme4 (version 1.1-26; Bates et al., 2015), lmerTest (version 3.1-3; Kuznetsova et al., 2017), and car (version 3.1-2; Fox & Weisberg, 2019) packages in R (R Core Team, 2021). Sum coding was used for the two-level contrasts of ISI (300ms = +1, 1000ms = -1) and condition (phonologically related= +1, unrelated real signs = -1; semantically related = +1, unrelated real signs = -1; English gloss rhyme = +1, unrelated real signs = -1). All analyses were conducted with a threshold for significance of alpha = .01. When the variable of AoA was replaced by age at testing or years of experience in the following models, there was no effect of either variable.

**Response Time**

We used linear-mixed effects modeling (lmer) to test the fixed effects of age of acquisition, ISI, and trial condition (unrelated, phonological, semantic, bilingual), along with interactions between the effects, on the continuous outcome variable of response time in correct trials, with random intercepts for participants and trial. Subjective frequency and subjective iconicity of target ASL signs were again included as covariates. As described above, sum coding was used for ISI and condition.

There were no main effects of age of acquisition, ISI, or any related conditions, nor any interactions between any of the variables (**Figure S3**). This suggests that repetition response time was not modulated by the related conditions.

**Figure S3**


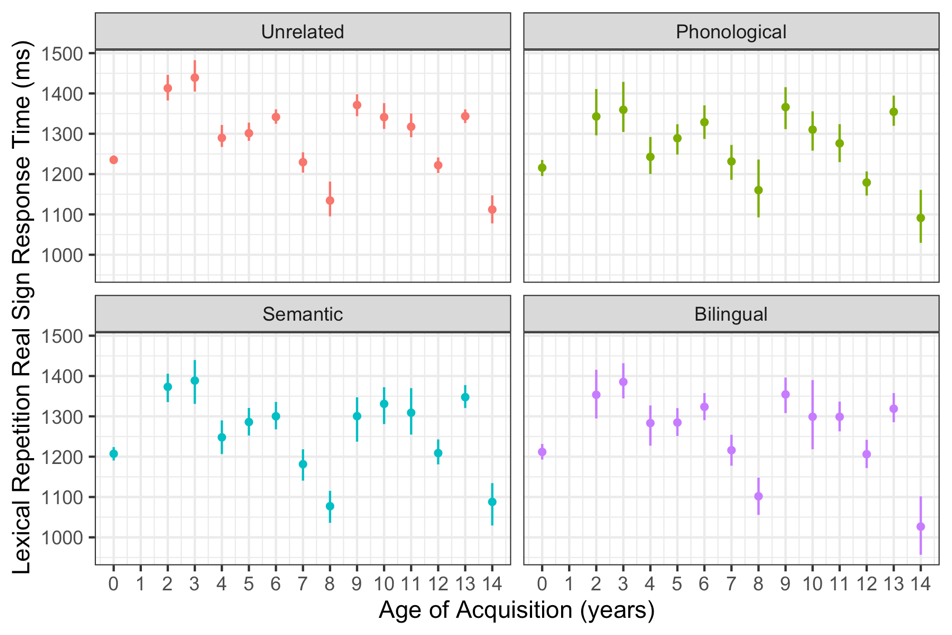


Lexical repetition response time by trial condition and age of acquisition with 95% confidence intervals.

**Production Time**

We used linear-mixed effects modeling (lmer) to test the fixed effects of age of acquisition, ISI, and trial condition (unrelated, phonological, semantic, bilingual), along with interactions between the effects, on the continuous outcome variable of production time in correct trials, with random intercepts for participants and trial. Subjective frequency and subjective iconicity of target ASL signs were included as covariates. As described above, sum coding was used for ISI and condition. For two participants, an error in gathering production time for a number of their trials resulted in excluding them from these analyses.

There were no main effects of age of acquisition, ISI, or any related conditions, nor any interactions between any of the variables (**Figure S4**). This suggests that repetition production time was not modulated by the related conditions.

**Figure S4**


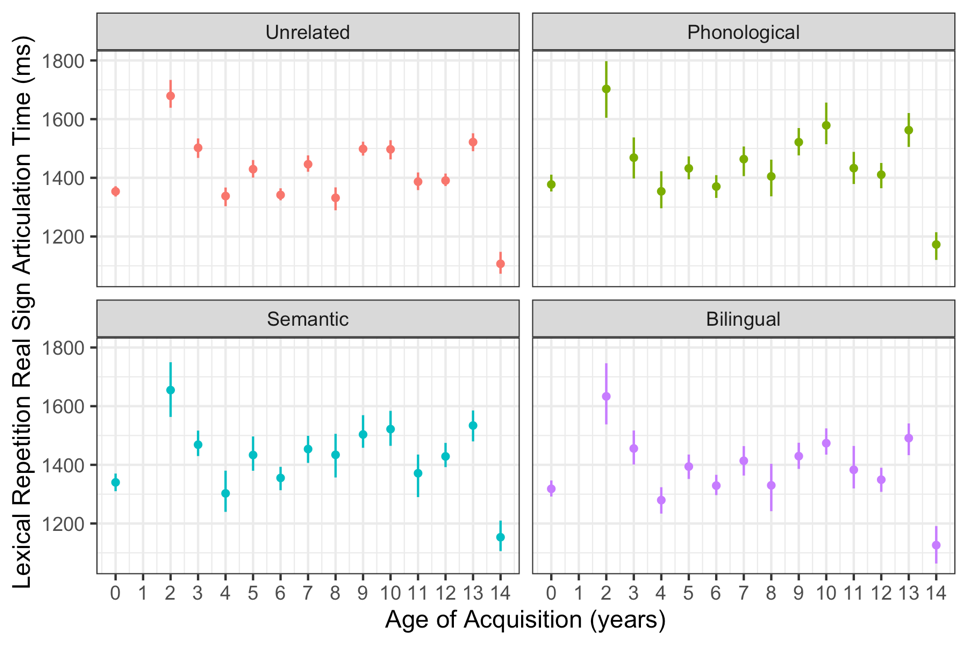


Lexical repetition production time by trial condition and age of acquisition with 95% confidence intervals.

**Accuracy**

We used generalized binomial linear-mixed effects modeling (glmer) to test the fixed effects of age of acquisition, ISI, and trial condition (unrelated, phonological, semantic, bilingual), along with interactions between the effects, on the binary variable of lexical repetition accuracy (0 = no error in repetition, 1 = at least one phonological error in repetition), with random intercepts for participants and trial. Subjective frequency and subjective iconicity of target ASL signs were again included as covariates. As described above, sum coding was used for ISI and condition.

Participants with later ages of acquisition made more repetition errors than those with earlier ages of acquisition (β = 0.091, SE = 0.020, z = 4.429, p < 0.001). There were no main effects of any of the related conditions or ISI, nor any interactions between any of the variables. This result is the same as in the main text showing that age of acquisition affected repetition accuracy (**Figure 6**), though with no effects of the related conditions (**Table S5**).

**Figure S5**


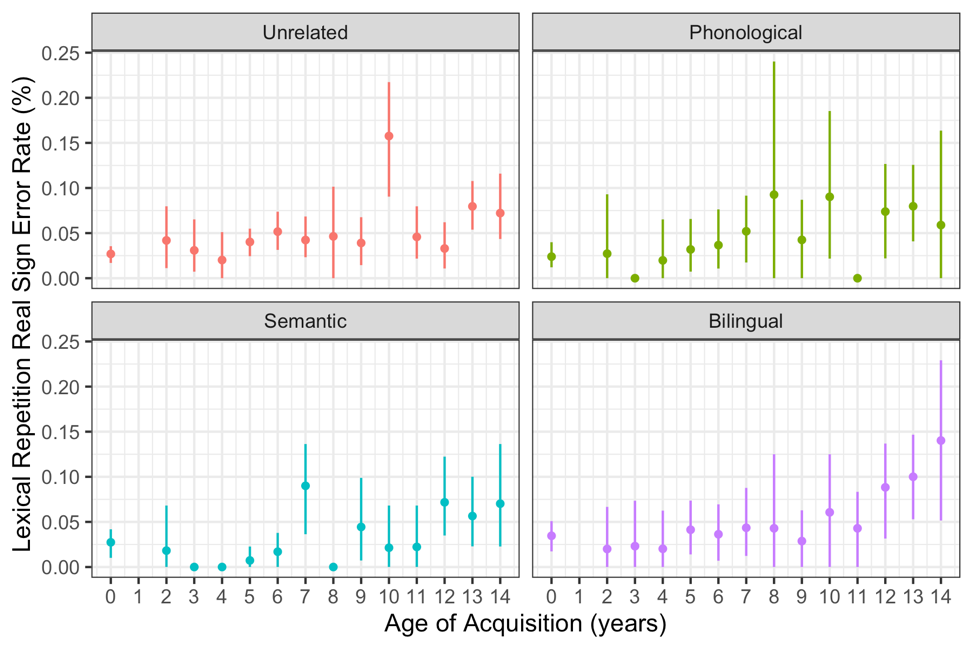


Lexical repetition error rate by trial condition and age of acquisition with 95% confidence intervals.

**Supplemental Discussion**

While there were three priming conditions, phonological, semantic, and bilingual (English gloss rhyme), we found no effects of any of these conditions in any analyses.

For the phonological related pairs, the lack of an effect may be caused by over-controlling the stimuli as there were only 24 total phonological priming trials and those were split even further to eight trials each for sharing handshape, sharing movement, or sharing location. As explained in the introduction, previous work suggests that each parameter contributes differently to the phonological system and each subsequently affects recognition differently with shared location showing inhibition effects (Baus et al., 2008; Carreiras et a., 2008; Corina & Hildebrandt, 2002), shared movement showing facilitation effects (Baus et al., 2014; Corina & Knapp, 2006; Dye & Shih, 2006), and shared handshape showing mixed effects (Carreiras et al., 2008; Corina & Emmorey, 1993; Corina & Hildebrandt, 2002; Dye & Shih, 2006). Because each parameter is proposed to differentially affect lexical recognition, it is possible that the attempt to examine all three together and keep the proportion of priming trials low did not provide sufficient power to detect phonological priming in this data set.

For the semantic condition, the lack of an effect may also be caused by a design flaw. This condition consisted of pairs of signs that were in an exemplar/subordinate relationship like [ROOM](https://asl-lex.org/visualization/?sign=room) and [BASEMENT](https://asl-lex.org/visualization/?sign=basement). After not seeing a priming effect in the semantic condition, we looked again at the stimuli in that condition and found that while some stimulus pairs were clearly in an exemplar/subordinate relationship, others may have been more like semantic associates, e.g., [WORK](https://asl-lex.org/visualization/?sign=work) and [SECRETARY](https://asl-lex.org/visualization/?sign=secretary), and others may have not been as clearly semantically related, e.g., [CLASS](https://asl-lex.org/visualization/?sign=class) and [HISTORY](https://asl-lex.org/visualization/?sign=history). It is possible that stronger and consistent semantic relationships would have elicited facilitation effects as seen in other research on the topic.

Lastly, the bilingual (English gloss rhyme) condition was used to investigate cross-language (cross-modal) activation. This has been well-attested for ASL and English in both directions (English to ASL and ASL to English; e.g., Lee et al., 2019; Meade et al., 2017; Morford et al., 2017) as well as bidirectionally in other spoken and sign languages like Chinese and Chinese Sign Language (Wang et al., 2022). Replicating the finding here would have provided further support of the existence of cross-language, cross-modal activation, but the lack of an effect is difficult to interpret. Cross-language activation across age of acquisition has not been investigated before and clearly more research is needed to determine whether later age of acquisition affects cross-language activation.

1. Subjective frequency ratings were taken from Mayberry et al. (2014), with the same participants as the present study. [↑](#footnote-ref-1)
2. Subjective iconicity ratings were not available for three of the target signs in the current study, so iconicity ratings for those three signs are not included in the analyses. [↑](#footnote-ref-2)
